# Systemic lupus erythematosus: updated insights on the pathogenesis, diagnosis, prevention and therapeutics

**DOI:** 10.1038/s41392-025-02168-0

**Published:** 2025-03-17

**Authors:** Xiaofeng Dai, Yuting Fan, Xing Zhao

**Affiliations:** 1 National Local Joint Engineering Research Center for Precision Surgery & Regenerative Medicine, Shaanxi Provincial Center for Regenerative Medicine and Surgical Engineering, First Affiliated Hospital of Xi’an Jiaotong University, Xi’an, 710061 P. R. China; 2 Tissue Engineering and Stem Cell Experiment Center, Tumor Immunotherapy Technology Engineering Research Center, Department of Immunology, College of Basic Medical Sciences, Guizhou Medical University, Guiyang, 550004 P. R. China; 3 Department of Gastroenterology, the Affiliated Hospital of Guizhou Medical University, Guiyang, 550001 P. R. China

**Keywords:** Rheumatic diseases, Adaptive immunity

## Abstract

Systemic lupus erythematosus (SLE) is a chronic inflammatory illness with heterogeneous clinical manifestations covering multiple organs. Diversified types of medications have been shown effective for alleviating SLE syndromes, ranging from cytokines, antibodies, hormones, molecular inhibitors or antagonists, to cell transfusion. Drugs developed for treating other diseases may benefit SLE patients, and agents established as SLE therapeutics may be SLE-inductive. Complexities regarding SLE therapeutics render it essential and urgent to identify the mechanisms-of-action and pivotal signaling axis driving SLE pathogenesis, and to establish innovative SLE-targeting approaches with desirable therapeutic outcome and safety. After introducing the research history of SLE and its epidemiology, we categorized primary determinants driving SLE pathogenesis by their mechanisms; combed through current knowledge on SLE diagnosis and grouped them by disease onset, activity and comorbidity; introduced the genetic, epigenetic, hormonal and environmental factors predisposing SLE; and comprehensively categorized preventive strategies and available SLE therapeutics according to their functioning mechanisms. In summary, we proposed three mechanisms with determinant roles on SLE initiation and progression, i.e., attenuating the immune system, restoring the cytokine microenvironment homeostasis, and rescuing the impaired debris clearance machinery; and provided updated insights on current understandings of SLE regarding its pathogenesis, diagnosis, prevention and therapeutics, which may open an innovative avenue in the fields of SLE management.

## Introduction

Systemic lupus erythematosus (SLE), canonically defined as an auto-immune disorder, can be considered as a chronic inflammatory illness with clinical manifestations encompassing various organs such as the blood vessels, brain, lungs, skin, kidneys and joints due to polymorphic biological alterations.^1^ It affects approximately 3.4 million people worldwide, with 400,000 individuals being newly diagnosed each year.^2,3^ It most commonly occurs among women between puberty and menopause,^4^ and individuals of the African origin have a higher risk of developing SLE.^5–7^ According to a 2023 global epidemiology study of SLE, Poland, the United States, Barbados, and China showed the highest SLE incidence.^2^ Though still with an unclear disease of origin, the chance of developing SLE is believed to be associated with genetic factors, epigenetic factors, environmental triggers, and hormonal factors.^8^

SLE can be diagnosed from the perspectives of disease onset and disease activity. These can be assessed using varied types of evaluation metrics such as the American College of Rheumatology (ACR) criteria and the SLE Disease Activity Index (SLEDAI). Besides, SLE is typically accompanied with increased risks of developing multiple types of comorbidities, with cancer screening being recommended by the European League Against Rheumatism (EULAR)^9^ and cerebrovascular disease being alarmed among female SLE patients by the American Heart Association.^10^

SLE is caused by an autoimmune reaction involving both the innate and adaptive immune systems, where an abnormal immune response is directed to nucleic acid-containing cellular particles. The over production of antibodies targeting these nucleic acids, known as antinuclear antibodies (ANAs), is characteristic of SLE.^8^ Besides, the anti-Smith (anti-Sm) antibody, which is an auto-antibody directed against a component of the spliceosome, is highly specific to SLE, with 20-40% SLE patients versus approximately 1% healthy individuals carrying them.^11^

Various types of agents have been used for SLE therapeutics, ranging from cytokines, antibodies, hormones, inhibitors, antagonists, to the transfusion of fresh plasma and stem cells. Importantly, while several drugs initially designed for treating other pathological conditions have been shown effective in treating SLE such as the use of the anti-malaria agent hydroxychloroquine as a SLE therapeutic, a plethora of medications have been reported capable of inducing SLE. Examples of this kind include anti-arrhythmic agents such as procainamide, broadspectrum antibiotics such as minocycline, vasodilators such as hydralazine and methyldopa, and antipsychotics such as chlorpromazine. These have unanimously complicated our understandings on the appropriate therapeutics of SLE, rendering it necessary and urgent to delve into the molecular mechanisms driving SLE pathogenesis and classify current therapeutics accordingly by their mechanisms-of-action. This may guide us towards effective management of SLE and, hopefully, help us identify the pivotal signaling axis for the establishment of innovative targeting strategies.

Following the introduction of some basic knowledge of SLE including the history and epidemiology, this review characterized three key determinants of SLE pathogenesis by their mechanisms-of-action, i.e., over-activated immune response, skewed cytokine microenvironment homeostasis, impaired debris clearance machinery; summarized current understandings on SLE diagnosis by disease onset, activity and comorbidity; introduced risk factors predisposing SLE at the genetic, epigenetic, hormonal and entrinsic levels; and classified current SLE preventive strategies and therapeutics by the identified working mechanisms. Our review not only provides comprehensive information on SLE so far available, but also proposes fresh insights on our current understandings of SLE, with a focus on its prevention and therapeutics.

## Basics of SLE

### Research history of SLE

**Fig. 1:**
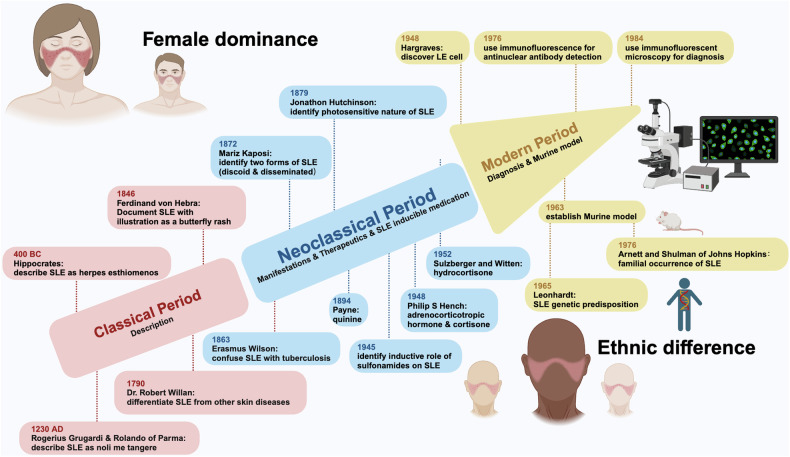
History and etiological features of SLE. The history of SLE studies is divided into three periods, i.e., classical period, neoclassical period, modern period. The classical period is characterized by ‘SLE description’, with critical events being ‘description of SLE as herpes esthiomenos by Hipocrates’, ‘description of SLE as noli me tangere by Rogerius Grugardi & Rolando of Parma’, ‘confusion of SLE with tuberculosis by Erasmus Wilson’, ‘description of SLE as skin disorders by Robert Willan’, and ‘differentiation of SLE from other diseases by Willan’. The neoclassical period is characterized by ‘SLE manifestions, therapeutics, and identification of the inductive role of some medications on SLE’, with representative events being ‘documentation of SLE with illustrations as a butterfly rash by Ferdinand Hebra’, ‘identification of the photosensitive nature of SLE by Jonathon Hutchison’, ‘definition of the two forms of SLE, i.e., discoid and disseminated SLE by Mariz Kaposi’, ‘use of quinine b Payne and use of adrenocorticotropic hormone (ACTH) and cortisone by Philip S. Hency’, ‘use of hydrocortisone by Sulzberger and Witten’, and ‘identificaton of the inductive role of sulfonamides on SLE’. The modern period is characterized by ‘SLE diagnosis and establishment of murine model’, with milestone events being ‘discovery of lupus erythematosus (LE) cell by Hargraves’, ‘use of immunofluorescence for antinuclear antibody (ANA) detection’, ‘use of immunofluorescent microscopy for diagnosis’, and ‘establishment of murine model’, where following murine model establishment, the genetic predisposition and familial occurrence of SLE were consecutively recognized

The history of SLE can be dated back to 400 Before Christ (BC) and divided into three periods, i.e., the classical period, the neoclassical period, and the modern period. The classical period, cornerstoned by Hipocrates who firstly described the possible ulcers of SLE as herpes esthiomenos, identified and documented SLE as a cutaneous disorder.^12^ The neoclassical period witnessed the manifestations and therapeutics of SLE. During this period, Ferdinand Hebra reported the facial rash associated with SLE as a butterfly rash with illustrations; and Jonathon Hutchison noted the photosensitive nature of SLE, among other milestones. Regarding SLE therapeutics, quinine was firstly used by the Physician Payne, followed by the use of adrenocorticotropic hormone and cortisone by the physician Philip S Hency, and later hydrocortisone by Sulzberger and Witten. In addition, the inductive role of medications such as sulfonamides on SLE was found during this period.^12^ The modern era was heralded by the discovery of the lupus erythematosus (LE) cell (a bone marrow phenomenon involving the phagocytosis of nuclear material by polymorphonuclear leukocytes) and characterized by rapid scientific advances over the past 60 years. Before the discovery of LE cell by Hargraves in 1948, the Wasserman test for syphilis was used for SLE diagnosis. The use of immunofluorescence for ANA detection followed. The establishment of the murine models has substantially advanced the scientific field related to SLE, leading to the discovery of the genetic predisposition of SLE by Leonhardt and the familial association of SLE by Arnett and Shulman of Johns Hopkins.^12^ Thanks to the contributions of these scientists made along the history of SLE researches, the lifespan of SLE patients has now been extensively extended from no longer than 5 years after the initial diagnosis to living with illness^12^ (Fig. 1).

### Epidemiology of SLE

SLE is a heterogeneous disease occurring frequently among women and least common among children.^8^ SLE most commonly afflicts women between puberty and menopause,^13^ where the female/male ratio shifts from 3/1 in children to about 9/1 or even 15/1 among adults between puberty and menopause^14,15^ (Fig. 1).

SLE is associated with an increased risk of premature mortality that has improved over the past 30 years,^16^ and the risk conveys an ethnic-dependent difference.^15,17^ The development of lupus nephritis (LN), a SLE-associated renal complication, is considered a strong predictor of an increased mortality risk. SLE patients of African, Chinese and Hispanic origins have shown an increased risk of developing LN^18,19^ and thus enhanced mortality.^18–20^ Besides mortality, the disease incidence, prevalence, age-of-onset, and morbidity of SLE also vary greatly among regions.^17^ For instance, the annual incidence and prevalence rates of SLE in the United States each varies from 2 to 7.6 and from 19 to 159 per 100 000 individuals, respectively, for people of different racial backgrounds.^5,6^ In particular, individuals of the African origin, particularly those who have migrated to America or Europe, exhibit a higher incidence and prevalence, earlier age at the disease-of-onset as compared with those of the north European origin^5–7^ (Fig. 1). Asian have a lower regional risk of developing SLE than people from the United States,^21,22^ but the prevalence of SLE among people carrying the Chinese background has been reported to be increasing.^23^ Such ethnic differences among SLE patients may be explained by their different socioeconomic backgrounds, distinct perceptions on the condition, varied risks of getting infection or developing comorbidities especially cerebrovascular diseases, imbalanced availability of the medical resources, and non-uniform adherence to the therapeutics.^3,16,17,24^

## SLE pathogenesis by mechanisms-of-action

SLE is characteristic of increased presentation of autoantibodies such as ANA, anti-Sm, anti-double-stranded DNA (anti-dsDNA) antibody, antiphospholipid (aPL) antibodies, and anti-β2-glycoprotein (aβ2GPI) antibodies,^1^ which even occur years prior to the clinical onset of SLE.^25^ Being the primary effectors of SLE inflammation and associated damage,^26^ autoantibodies form immune complexes (ICs) and deposit on multiple organs such as kidney, skin and central nervous system to induce local inflammation.^26,27^ Antibodies are produced by plasma cells (PCs) and plasmablasts, the terminally differentiated B cells.^28^ As the precursors of PCs and important antigen-presenting cells (APCs),^29,30^ B cells loose tolerance to autoantigens^31^ and may present them to T cells in SLE patients followed by the activation of Th cells. Stimulated Th cells activate B cells and contribute to their differentiation through clusters of differentiation 40 ligand (CD40L)/CD40 interactions.^32^ Activated B cells move to germinal centers (GCs) with the help of Tfh cells and follicular dendritic cells (DCs), where B cells generating antibodies with high antigen affinity are expanded and differentiate into the memory B cells (MBC) and antibody-producing PCs.^33^

### Over-production of immunoactivating materials

Interferons (IFNs) are cytokines with pleiotropic roles in immune regulation that can be categorized into type I, II, and III based on sequence homology.^34^ Type I IFNs represent the largest IFN family comprised of IFNα, IFNβ, IFNω, IFNκ, and IFNε; the type II family includes solely IFNγ; and the type III family contains IFNλ1, IFNλ2, IFNλ3, and IFNλ4.^35^ Out of the three families of IFNs, type I IFNs play an immunomodulatory role that bridges the gap between the innate and adaptive immune systems.^35^ That is, the expression of type I IFNs is activated on the trigger of nucleic acids via intracellular pathways such as Toll-like receptor (TLR)-mediated signaling; the binding of type I IFNs to their receptors activates the intracellular signaling cascade involving the Janus kinase-signal transducer and activator of transcription (JAK/STAT) axis that leads to stimulated effectors of the innate and adaptive systems.^35–37^

There has been a well-established positive association between type I IFNs and SLE. Specifically, the blood levels of type I IFNs were documented to be elevated in approximately 50% of SLE patients.^38^ An even greater percentage of SLE patients were estimated to carry over-represented expression of genes involved in type I IFN-mediated signaling in their peripheral blood cells.^39,40^

Type I IFNs are generated in response to, primarily, the activation of nucleic acid-binding pattern recognition receptors such as the endosomal TLR3/4/7/9, the cytosolic sensor cyclic guanosine monophosphate-adenosine monophosphate (cGMP-AMP) synthase (cGAS), and the ribonucleic acid (RNA)-sensor retinoic acid-inducible gene (RIG) I like receptors (RLRs)-mitochondreal antiviral signaling protein (MAVS).^41^ These nucleic-acid sensing pathways are chronically over-activated in many SLE patients, with the pathogenesis roles of TLR7 in SLE being well-established.^42^ For instance, over-activation of the cGAS-stimulator of IFN genes (STING) pathway has been shown to be crucial in autoimmunity and SLE pathogenesis.^40,43^

**Fig. 2:**
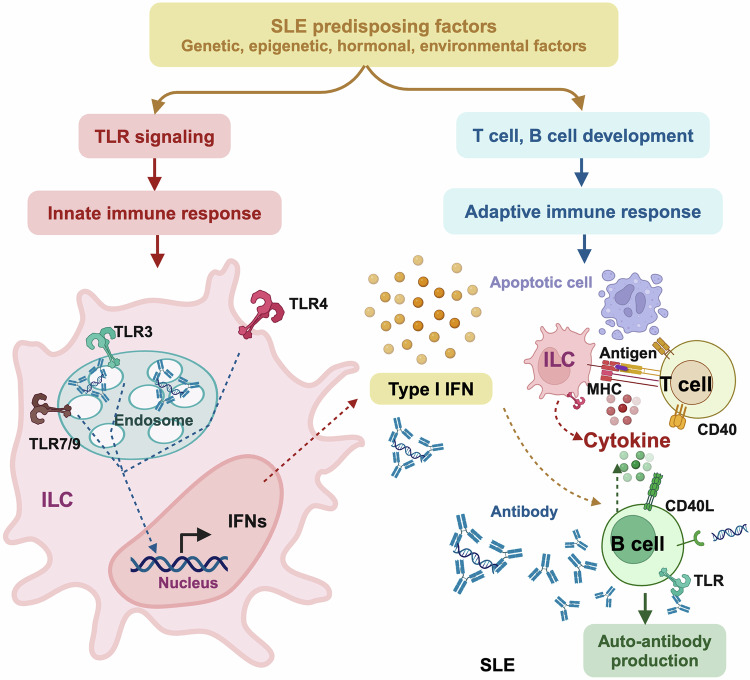
Mechanisms-of-action driving SLE pathogenesis via over-activating immune response. SLE predisposing factors including genetic, epigenetic, hormonal and environmental factors can function as or induce the production of immunoreactants to trigger Toll-like receptor (TLR) signaling in the innate immune system for enhanced generation of type I interferon (IFN), or modulate the development and maturation of T and B cells in the adaptive immune system for over-production of auto-antibodies. Type I IFN functions as the hub bridging the innate and adaptive immune systems. Specifically, type I IFN is produced from innate lymphoid cells (ILC), and directly triggers B cell activation to overtly produce auto-antibodies that ultimately contribute to SLE pathogenesis. Besides producing type I IFNs, ILCs can also present antigens to T cells via major histocompatibility complex (MHC) to activate T cells that express CD40L that interacts with B cells via the CD40L-CD40 bridge for enhanced auto-antibody production

In addition, events altering nucleic acid metabolism may also trigger type I IFNs production, where the essential roles of cytosolic nucleic acid sensors played in SLE have been well characterized.^44,45^ For instance, ultraviolet (UV) light exposure has been shown capable of enhancing type I IFNs response both locally and systemically, with the evidence being obtained from both the animal model and from the clinics^46^ (Fig. 2).

Type I IFNs are central to the activation of both the innate and the adaptive immune systems. Specifically, by interacting with their receptors, type I IFNs induce signaling through the JAK/STAT pathway followed by the transcription of IFNs-responsive genes that encode the ‘IFN signature’ for activated immune response. On the other hand, type I IFNs can directly activate DCs for enhanced presentation of antigens to T cells, where T cell activation and polarization are important to prime B cell differentiation. Activated B cells then produce auto-antibodies that, once over-produced without timely clearance, deposit in organs and cause tissue damages, the process of which is facilitated by the interactions between CD40 and CD40L^40^ (Fig. 2).

### Skewed cytokine microenvironment

There are two types of T cells, i.e., αβ^+^ and γδ^+^ T cells, and αβ^+^ T cells can be further classified into CD4^+^ and CD8^+^ T cells. While CD8^+^ T cells take on the cell-killing activity,^47^ CD4^+^ T cells are T helper (Th) cells capable of promoting CD8^+^ T cell development and B cell differentiation as well as antibody synthesis.^48,49^ CD4^+^ T cells can be roughly subdivided into Th1, Th2, Th17 and regulatory T (Treg) cells, based on their distinct cytokine profilings.^50^ Th1 cells produce, primarily, IFNγ, interleukin (IL)12, IL2, tumor necrosis factor (TNF) alpha, IL1β, granulocyte-macrophage colony-stimulating factor (GM-CSF); Th2 cells secrete IL4, IL5, IL6, IL10, IL13, among others;^51^ Th17 cells are featured by expressing IL17A, IL17F and IL22;^52^ and Tregs secrete transforming growth factor beta (TGFβ), IL10, IL34, IL35 etc.^53,54^ These cytokines form the cytokine microenvironment to support the amplification of a self-directed immune response, perturbed homeostasis of which may lead to disease syndromes including SLE.

With our incremental understandings on the heterogeneity of Th cells and their roles in regulating cytokine homeostasis, more subcohorts of Th cells such as T follicular helper (Tfh), T peripheral helper (Tph), follicular regulatory T (Tfr) cells have been consecutively identified, some of which hold essential roles in SLE.^55–59^ For instance, expansion of Tfh and Tph is a prominent feature of SLE,^58^ and Tfr cells can suppress B cell activation via disrupting the recognition and interaction between Tfh cells and B cells.^55^ B cells have been previously considered to be stimulated by Th2 cells but are now considered primed by Tfh cells. Thus, Tfh and Tph function in the Th2 linkage towards activated B cells and enhanced SLE severity, Tfr cells act as the switch-off button suppressing the activity of Tfh.^60^ We focus on the Th1/Th2 and Treg/Th17 pairs that build up the conceptual framework controlling cytokine homeostasis, where T cell subsets involved in fine-gained regulations within this framework such as the modulatory roles of Tfh were not comprehensively covered or thoroughly discussed here.

Besides CD4^+^ and CD8^+^ T cells, double-negative (DN) T cells (a unique subset of T cells lacking both CD4 and CD8 co-receptors) also play a significant role in SLE pathogenesis. DN T cells are formed by T lymphoid progenitor cells never traveled through thymus, by T lymphoid progenitor cells traveled through thymus but lack further development into CD4^+^ or CD8^+^ T cells, and by CD4^+^ or CD8^+^ T cells with down-regulated CD4 or CD8 co-receptor.^61^ DN T cells comprise 1-3% of human T cells and are of high heterogeneity. There exists at least five DN T cell cohorts, i.e., helper DN, cytotoxic DN, innate DN, resting DN, and intermediate DN.^62^ DN T cells may be pro-inflammatory such as IL17-producing DN T cells^63^ and anti-inflammatory such as IL10-producing DN T cells.^64^ The roles so far reported on DN T cells in SLE are largely pro-inflammatory. Specifically, the amount of DN T cells was considered to be positively correlated with SLE activity;^65,66^ DN T cells conveyed a positive impact on B cell-mediated antibody production by stimulating the release of IL4, IL17 and IFNγ;^67^ and enhanced DN T cell apoptosis as a result of inhibited neddylation attenuated lupus progression in murine models.^66^ Though the significance of DN T cells in SLE pathogenesis is not negligible, they are exempted from the focus of this section given the shared cytokines (such as IL4, IL17 and IFNγ) they produce with different CD4^+^ T subsets, complex functional plasticity (having both helper and cytotoxic DN cohorts) and small percentage.

#### Th1/Th2 imbalance

**Fig. 3:**
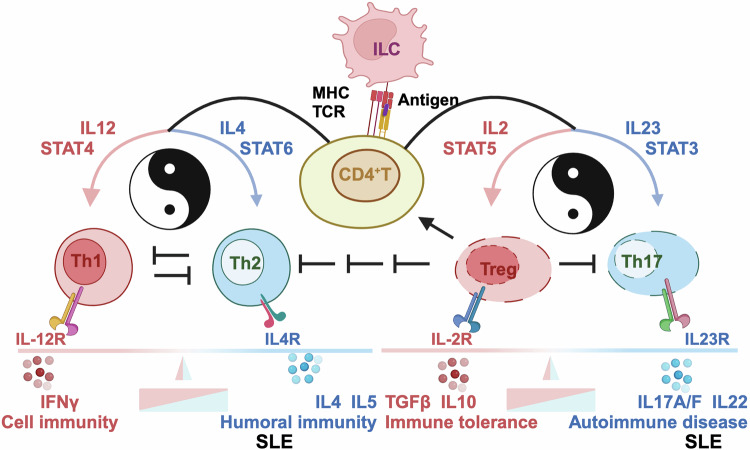
Mechanisms-of-action driving SLE pathogenesis via skewing cytokine microenvironment. On antigen presentation to CD4 + T cells through T cell receptor (TCR) by innate lymphoic cells (ILC) via major histocompatibility complex (MHC) from the innate immune system, CD4^+^ T cells are activated and can differentiate into distinct T cell subsets such as T helper 1 (Th1), T helper 2 (Th2), T helper 17 (Th17) and T regulatory (Treg) cells, the direction of which is dictated by the cytokine milieu of the microenvironment. Specifically, when IL12 is enriched in the environment, T cells favor Th1 polarization, the process of which involves STAT4 signaling that leads to up-regulated IFNγ and down-regulated IL4/5; when IL4 is enriched in the milieu, T cells are triggered for Th2 clonal expansion via STAT6, resulting in up-regulated IL4/5 expression and down-regulated IFNγ level. On the other hand, IL23, among others, drives T cell plasticity towards Th17 phenotype, the process of which involves STAT3 signaling; and IL2, out of other cytokines, inhibits Th17 generation but promotes Treg generation, where STAT5 plays a role. The balances between Th1 and Th2 cells dictates the preference towards cell immunity and humoral immunity, respectively, as regulated by IFNɤ and IL4/5, respectively. The homeostasis between Treg and Th17 cells determines whether the system goes for immune tolerance (that can cause chronic infectious diseases including cancers) or immune activation (that can lead to the pathogenesis of autoimmune diseases including SLE), as regulated by TGFβ, IL10 (among other cytokines produced by Treg cells) and by IL17A, IL17F, IL22 (out of other cytokines generated by Th17 cells). These subsets of CD4^+^ T cells cross-regulate among themselves. In particular, Th1 and Th2 cells suppress the expression of each other, Treg cells reduce the levels of Th1, Th2, Th17 cells, and activates that of CD4^+^ T cells. Only representative cytokines are listed in this Figure. that do not exclude the existence of others

Cytokines produced by Th1 cells are largely pro-inflammatory, and those generated by Th2 cells are primarily anti-inflammatory.^68^ Numerous evidence has suggested that abnormal T cell differentiation to Th2 dominance can lead to B cell hyper-activation that contributes to immune disorders including SLE pathogenesis.^51^ The selective development of Th1 and Th2 cells is primarily driven by the cytokine microenvironment among other influential factors such as antigen dose, affinity of antigens, major histocompatibility complex (MHC) haplotypes and co-stimulatory factors. Among other cytokines, IL12 and IL4 dictate the fate of Th cells to the Th1 or Th2 linkage, respectively. While IL12 drives Th1 cell differentiation through STAT4 signaling that leads to up-regulated IFNγ and down-regulated IL4/IL5 for amplified Th1 proliferation, IL4 induces Th2 clonal expansion through STAT6 that results in up-regulated levels of IL4/IL5 and down-regulated IFNγ expression for augmented Th2 differentiation (Fig. 3).^69^

#### Th17/Treg imbalance

The imbalance between pro-inflammatory Th17 cells and immuno-suppressive Tregs underlies the pathogenesis of SLE.^70–72^ The proportion of Th17 cells is higher in SLE patients, the content of which is positively correlated with SLE severity.^73^ Tregs play an important role in maintaining the immune tolerance, reduced levels or activities of which are tightly associated with the onset and progression of SLE^74–76^ (Fig. 3).

The subsets of Th17 cells and Tregs have distinct metabolic patterns. While Th17 cells are mainly powered by glycolysis, the energy supply of Tregs largely relies on fatty acid oxidation and oxidative phosphorylation.^77^ Accordingly, glycolysis deprivation was found to impair Th17 cell differentiation but support the growth of Tregs;^78–81^ and impaired fatty acid oxidation was associated with increased Th7 cell linkage yet diminished Tregs development.^82^ Thus, switching energy supply from relying on carbohydrates to lipids (low-carb or ketogenic-diet) may be a dietary recommendation for SLE patients.

### Impaired debris clearance machinery

The pathogenesis of SLE is associated with the failure of removing self-reactive clones of T and B cells. Under normal conditions, an immune response against self-antigens (i.e., anergic responses) can be suppressed by the immune system; however, when this debris clearance machinery is impaired, the ICs (comprised of, e.g., nucleic acids, nucleic acid-binding proteins, autoantibodies directed against those components) may form and initiate the onset of inflammation and organ damage; perpetuation of damage occurs when the ICs further amplifies the immune system followed by the trigger of downstream signals that induce pro-inflammatory mediators such as IFNα, leading to or aggregating the pathogenesis of SLE.

**Fig. 4:**
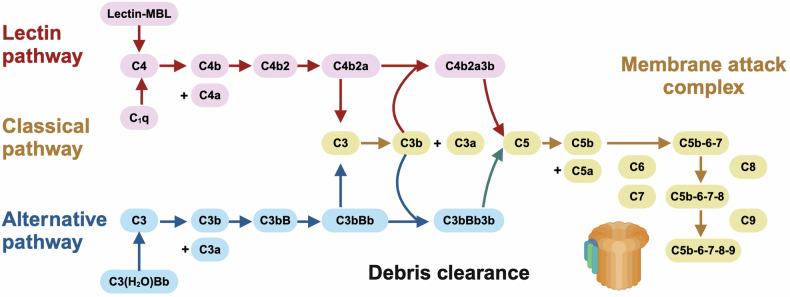
Mechanisms-of-action driving SLE pathogenesis via impairing debris clearance machinery. There are three mechanisms to initiate the complement cascade for debris clearance, i.e., the classical, lectin, and alternative pathways. In the classical and lectin pathways, the complement system is triggered by the binding of the antibody complexes to C1q of the C1 complex and the binding of foreign carbohydrate moieties to mannose binding lectin (MBL) or ficolin, respectively, which converge to the cleavage of C4 to C4b and C4a followed by the generation of one C3 convertase, i.e., C4b2a. In the alternative path, the complement system is activated via spontaneous hydrolysis of C3 into C3b and C3a by the convertase C3(H2O)Bb followed by the formation of another C3 convertase, i.e., C3bBb. Under the cleavage of C4b2a or C3bBb, C3 becomes C3b and C3a. Following this, C3b binds to C4b2a or C3bBb to form C4b2a3b or C3bBbC3b which are C5 convertases capable of hydrolyzing C5 into C5b and C5a; and C5b can initiate the cascade of forming the membrane attack complex (MAC) capable of generating pores in the membranes of pathogens or targeted cells. While C3 activation fragments such as C3b participate in the cleaning of cellular debris to avoid overt activation of the immune system that is favorable for halting SLE pathogenesis, overt production of MAC may promote SLE pathogenesis via causing cell death and generating more immunoreactants

The complement system is centered at the core of the immune system mediating a cross-talk between the innate and adaptive immune responses. The complement system has been implicated in diverse biological processes in mammals including, e.g., modulation of the immune tolerance, and autoimmune diseases. Dynamic homeostasis between the activation and inhibition of the complement system is required to maintain human health. While hyper-activated complement system may lead to excessive inflammation and tissue damage, hypo-activation of the complement machinery may impair debris clearance and lead to autoimmune disorders including SLE^83^ (Fig. 4).

The complement cascade is activated to initiate the proteolytic cleavage of complement proteins into fragments that relay signals to neighboring cells and leukocytes by being deposited onto the targets or released into the extracellular fluid. There are three mechanisms, so far elucidated, to activate the complement system, i.e., the classical, alternative, and lectin pathways.^83^ In the classical pathway, the complement system is triggered by the binding of the antibody complexes to C1q of the C1 complex. In the alternative path, the complement system is activated via spontaneous hydrolysis of C3. In the lectin pathway, the system is stimulated via the binding of foreign carbohydrate moieties to mannose binding lectin (MBL) or ficolin. All pathways converge to the generation of C4b2a or C3bBb, the cleavage of C3, and the amplification loop. C3b, cleaved from C3, then promotes the formation of C4b2a3b in the classical/lectin pathways or C3bBb3b in the alternative pathway. C4b2a3b or C3bBb3b activate C5 to form the membrane attack complex (MAC) that takes on the action through direct lysis of the target cells. Perturbed activation or availability of any part of this cascade may impair immune homeostasis and lead to severe clinical syndromes such as SLE. For instance, over-expression of complement C3 has been associated with promoted gastric cancer progression via activating JAK2/STAT3 signaling^84^ (Fig. 4).

Besides the complement system, other mechanisms also exist for debris clearance. For instance, marginal zone macrophages (MZMs) are crucial for clearing apoptotic cells and maintaining immune tolerance, dysfunction of which can lead to defected clearance of apoptotic cells, activated autoreactive T cells and expanded DN T cells.^63^

## Diagnosis of SLE

### Disease onset

SLE is highly heterogeneous with variable distinct clinical manifestations, and the disease severity varies from mild to moderate and to severe. For instance, skin inflammation might be restricted to malar rash in one individual but involve upper extremity and trunk as well in another.

The diagnosis of SLE is challenging as no consensus has been made on the diagnostic criteria that needs to be of both a high specificity and a high sensitivity.^85^ Currently, SLE is diagnosed by both clinical manifestations and laboratory examinations, where SLE manifestations can be defined by the presence of both subjective and objective findings, as well as laboratory examinations. Subjective observations include, e.g., headaches, chest pains, and arthralgias. Objective documentations include, e.g., electrocardiographic or echocardiographic confirmation of cardiac comorbidities. Lab tests include, e.g., autoantibody detection, functional test and imaging.^8^

The 1997-version ACR classification criterion has been canonically used for SLE diagnosis,^86,87^ with the classification indexes used being malar rash, discoid rash, photosensitivity, oral ulcers, non-erosive arthritis, pleuritis or pericarditis, renal disorder, neurological disorder, hematological syndrome, immunological evidence, and positive ANA. In the clinical practice, malar rash refers to erythema over the malar eminences that tends to spare the nasolabial folds; discoid rash is defined as erythematous raised patches with adherent keratotic scaling and follicular plugging; photosensitivity is documented as skin rash on sunlight exposure; oral ulcers is considered as oral or nasopharyngeal ulceration that is typically painless; non-erosive arthritis is defined as tenderness, swelling or effusion in peripheral joints; pleuritis or pericarditis refers to rubbing or evidence of pleural/pericardial effusion; renal disorder is considered if persistent proteinuria of >0.5 g/day occurred or cellular casts were present in urine including red blood cells or hemoglobin; neurological disorder primarily refers to seizures or psychosis; hematological disorder refers to hemolytic anemia with reticulocytosis, leukocytopaenia, lymphocytopaenia, or thrombocytopaenia; immunological evidence refers to the presence of anti-deoxyribonucleic acid (DNA) autoantibody, anti-Sm autoantibody, or aPL autoantibodies; and positive ANA is defined as abnormal presence of ANA.^8^ These 11 indexes were updated in the 2019 EULAR/ACR classification criterion, with ‘fever’, ‘autoimmune hemolysis’, ‘non-scarring alopecia’, ‘low complement levels of C3 and/or C4’ being included, and ‘malar rash’ and ‘photosensitivity’ being removed. In addition, positive ANA was considered as an entry criterion for SLE characterization and a weighted system was used to assess the clinical and immunological manifestations of SLE in the 2019 EULAR/ACR classification criterion.^88,89^ Though specificities of these two ACR versions remain similar (i.e., 93%), the sensitivity of the 2019-version improved from 83% to 96% as compared with the 1997-version^88^ (Table 1).

**Table 1:** Current criteria and indexes for SLE diagnosis

Criteria	Application		Index	Annotation	References
ACR criteria (1997)	Onset	Cardiopulmonary	Pluritis or pericarditis	Rubbing or evidence of pleural/pericardial effusion	^86,87^
		Hematological	Hemolytic anemia	Hemolytic anemia with reticulocytosis.	
			Leukocytopaenia	<4000/mm^3^ on two or more occasions in the absence of causative drugs.	
			Lymphocytopaenia	<1500/mm^3^ on two or more occasions in the absence of causative drugs.	
			Thrombocytopaenia	<100,000/mm^3^ in the absence of causative drugs.	
		Immunologic	Positive anti-nuclear autoantibody	Abnormal titer of antinuclear antibody by immunofluorescence or an equivalent assay at any point intime and in the absence of drugs known to be associated with drug-induced lupus’ syndrome	
			Positive anti-DNA autoantibody	Positive antibody to native DNA in abnormal titer.	
			Positive anti-Sm	Presence of antibody to Sm nuclear antigen.	
			Positive anti-phospholipid antibody	(1) an abnormal serum level of IgG or IgM anticardiolipin antibodies, (2) a positive test result for lupus anticoagulant using a standard method, or (3) a false-positive serologic test for syphilis known to be positive for at least 6 months and confirmed by Treponema pallidum immobilization or fluorescent treponemal antibody absorption test	
		Mucocutaneous	Discoid rash	Erythematous raised patches with adherent keratotic scaling and follicular plugging.	
			Malar rash	Erythema over the malar eminences that tends to spare the nasolabial folds.	
			Oral ulcers	Oral or nasopharyngeal ulceration that is typically painless.	
			Photosensitive rash	Skin rash on sunlight exposure.	
		Musculoskeletal	Non-erosive arthritis	Tenderness, swelling or effusion in peripheral joints.	
		Neuropsychiatric	Psychosis	A mental state characterized by a disconnection from reality, often involving hallucinations or delusions, in the absence of offending drugs or known metabolic derangements such as uremia, ketoacidosis or electrolyte imbalance.	
			Seizures	Sudden, uncontrolled electrical disturbances in the brain that can cause changes in one's behavior, movements, feelings, and levels of consciousness, in the absence of offending drugs or known metabolic derangements such as uremia, ketoacidosis or electrolyte imbalance.	
		Renal	Proteinuria and/or cellular casts	Persistent proteinuria of >0.5 g/day occurred, or cellular casts were present in urine including red blood cells or hemoglobin.	
SLICC criteria (2012)	Onset	Cardiopulmonary	Pleuritis or pericarditis	(1) Typical pleurisy for more than 1 day or pleural effusions or pleural rub, and (2) typical pericardial pain (pain with recumbency improved by sitting forward) for more than 1 day or pericardial effusion or pericardial rub or pericarditis by electrocardiography (in the absence of other causes, such as infection, uremia, and Dressler’s pericarditis).	^92^
		Hematological	Hemolytic anemia	Present of anemia, reticulocytosis, low haptoglobin, high lactate dehydrogenase, and high indirect bilirubin.	
			Leucopenia	White blood cells <4000/mm^3^ at least once.	
			Thrombocytopenia	Platelet <100,000/mm^3^ at least once.	
		Immunologic	Positive dsDNA autoantibody	Anti-dsDNA antibody level above laboratory reference range (or 2-fold the reference range if tested by ELISA).	
			Positive anti-nuclear autoantibody	Anti-nuclear autoantibody level above laboratory reference range	
			Positive anti-phospholipid antibody	Positive test result for lupus anticoagulant, false-positive test result for rapid plasma regain, medium- or high-titer anticardiolipin antibody level (IgA, IgG, or IgM). Positive test result for anti-β2-glycoprotein I (IgA, IgG, or IgM).	
			Positive anti-Sm	Presence of antibody to Sm nuclear antigen	
			Positive direct Coombs' test	Direct Coombs’ test in the absence of hemolytic anemia.	
			Low complement	Low C3, low C4, low 50% hemolytic complement.	
		Mucocutaneous	Acute cutaneous lupus	(1) Lupus malar rash (do not count if malar discoid), bullous lupus, toxic epidermal necrolysis variant of SLE, maculopapular lupus rash, photosensitive lupus rash, in the absence of dermatomyositis, or (2) subacute cutaneous lupus (nonindurated psoriaform and/or annular polycyclic lesions that resolve without scarring, although occasionally with postinflammatory dyspigmentation or telangiectasias).	
			Chronic cutaneous lupus	Classic discoid rash, localized (above the neck), generalized (above and below the neck), hypertrophic (verrucous) lupus, lupus panniculitis (profundus), mucosal lupus, lupus erythematosus tumidus, chillblains lupus, discoid lupus/lichen planus overlap.	
			Non-scarring alopecia	Diffuse thinning or hair fragility with visible broken hairs (in the absence of other causes such as alopecia areata, drugs, iron deficiency, and androgenic alopecia).	
			Oral ulcers	Palate, buccal, tongue or nasal ulcers (in the absence of other causes, such as vasculitis, Behcet’s disease, infection (herpesvirus), inflammatory bowel disease, reactive arthritis, and acidic foods).	
		Musculoskeletal	Synovitis	Swelling or effusion or tenderness in 2 or more joints and at least 30 minutes of morning stiffness.	
			Myelitis	Damage to the spinal cord, which can cause weakness, pain, and other neurological symptoms.	
		Neuropsychiatric	Delirium	(1) Change in consciousness, or (2) level of arousal with reduced ability to focus, and (3) symptom development over hours to <2 days, and (4) symptom fluctuation throughout the day and either (4a) acute/subacute change in cognition (e.g., memory deficit or disorientation) or (4b) change in behavior, mood or affect (e.g., restlessness, reversal of sleep/wake cycle and so on) in the absence of other causes, including toxic/metabolic, uremia, drugs.	
			Mononeuritis multiplex	A neurological condition characterized by the inflammation of multiple individual nerves simultaneously, in the absence of other known causes such as primary vasculitis.	
			Peripheral neuropathy	A form of Guillain-Barré syndrome, where there is rapid onset of inflammation and damage to multiple peripheral nerves. Damage or dysfunction affecting one or more of the 12 pairs of cranial nerves, leading to a range of symptoms affecting many parts of the body in the absence of other known causes such as primary vasculitis, infection, and diabetes mellitus	
			Psychosis	(1) Delusions and/or hallucinations without insight, and (2) absence of delirium.	
			Seizures	Primary or generalised seizure or partial/focal seizure, with independent description by a reliable witness. If electroencephalography is performed, abnormalities must be present.	
		Renal	Protein-to-creatinine ratio	Urine protein-to-creatinine ratio (or 24-h urine protein) representing 500 mg protein/24 h or red blood cell casts.	
EULAR/ACR criteria (2019)	Onset	Cardiopulmonary	Acute pericarditis	≥2 of: (1) pericardial chest pain (typically sharp, worse with inspiration, improved by leaning forward), (2) pericardial rub, (3) ECG with new widespread ST-elevation or PR depression, (4) new or worsened pericardial effusion on imaging (such as ultrasound, X-ray, CT scan, MRI).	^89^
			Pleuritis or pericarditis	Imaging evidence (such as ultrasound, X-ray, computed tomography scan, magnetic resonance imaging) of pleural or pericardial effusion or both.	
		Constitutional	Fever	>38.3 °C with no other source identified.	
		Hematological	Autoimmune hemolysis	(1) Evidence of hemolysis, such as reticulocytosis, low haptoglobin, elevated indirect bilirubin, elevated LDH, and (2) positive Coomb’s (direct antiglobulin) test.	
			Leucopenia	White blood cells <4000/mm^3^ at least once.	
			Thrombocytopenia	Platelets<100 000/mm.^3^	
		Immunologic	Low complement	C3 and/or C4 below normal lower range.	
			Positive anti-nuclear autoantibody	A history of a positive anti-nuclear autoantibody by Hep 2 immunofluorescence ≥1:80.	
			Positive anti-dsDNA autoantibody, anti-Sm antibodies	Positive result for anti-dsDNA and/or anti-Smith antibodies.	
			Positive anti-phospholipid antibody	Anticardiolipin antibodies at medium or high titer or positive anti-β2-glycoprotein I antibodies or positive lupus anticoagulant.	
		Mucocutaneous	Acute cutaneous lupus	Malar rash (localised) or maculopapular rash (generalised) observed by a clinician, with or without photosensitivity. If skin biopsy is performed, typical changes must be present.	
			Discoid lupus	Discoid lupus is characterised by erythematous-violaceous cutaneous lesions with secondary changes of atrophic scarring, dyspigmentation, often follicular hyperkeratosis/plugging (scalp), observed by a clinician, leading to scarring alopecia on the scalp. Lesions have a preference for the head and neck, especially the conchal bowl, but may be found in nearly any location. If skin biopsy is performed, typical changes must be present.	
			Non-scarring alopecia	Diffuse thinning or hair fragility with visible broken hairs.	
			Oral ulcers	Oral or nasopharyngeal ulceration, usually painless and based on physician examination.	
			Subacute cutaneous lupus	Subacute cutaneous lupus is characterised by annular or papulosquamous (psoriasiform) cutaneous eruption observed by a clinician, usually photodistributed. If skin biopsy is performed, typical changes must be present.	
		Musculoskeletal	Synovitis	≥2 joints: characterised by joint swelling and tenderness, observed by a clinician.	
		Neuropsychiatric	Delirium	(1) Change in consciousness, or (2) level of arousal with reduced ability to focus, and (3) symptom development over hours to <2 days, and (4) symptom fluctuation throughout the day and either (4a) acute/subacute change in cognition (e.g., memory deficit or disorientation) or (4b) change in behavior, mood or affect (e.g., restlessness, reversal of sleep/wake cycle and so on).	
			Psychosis	(1) Delusions and/or hallucinations without insight, and (2) absence of delirium.	
			Seizures	Primary or generalised seizure or partial/focal seizure, with independent description by a reliable witness. If electroencephalography is performed, abnormalities must be present.	
		Renal	Lupus nephritis	Class I minimal mesangial LN Class II mesangial proliferative LN Class III focal LN Class IV diffuse segmental (IV-S) or global (IV-G) LN Class V membranous LN Class VI advanced sclerosing LN	
			Proteinuria	>0.5 g/24 h: on 24 h urine collection or spot urine protein-to-creatinine ratio representing >0.5 g protein/24 h.	
BILAG-2004	Activity	Cardiopulmonary	Aortitis	Inflammation of the aorta, the body's main artery, which can weaken its walls and cause complications.	^101^
			Arrhythmia	Irregular heartbeats, which can be too fast, too slow, or irregular, affecting the heart's ability to pump blood effectively.	
			Coronary vasculitis	Inflammation of the blood vessels supplying the heart, which can restrict blood flow and lead to heart problems.	
			Cardiac tamponade	Fluid accumulates around the heart, compressing it and impairing its function.	
			Endocarditis	Inflammation of the heart's inner lining, which can lead to heart failure if not treated.	
			Lupus peritonitis	Lupus associated inflammation of the peritoneum, the lining of the abdominal cavity.	
			Myocarditis	Inflammation of the heart muscle, which can affect its function and cause various symptoms.	
			Pleural effusion with dyspnea	Accumulation of fluid in the space between the lungs and chest wall, causing shortness of breath	
			Pleuritis or pericarditis	Pleuritis is defined by a convincing history of pleuritic pain, rubbing heard by a physician or evidence of pleural effusion. Pericarditis is documented by an electrocardiogram, rubbing heard by a physician or evidence of pericardial effusion	
			Pulmonary vasculitis or hemorrhage	Bleeding in the lungs or inflammation of the blood vessels in the lungs, affecting lung function.	
			Pneumonitis	Inflammation of the alveoli and the tissue surrounding them, causing difficulty breathing.	
			Shrinking lung	A condition where the lungs become smaller and stiffer, reducing their ability to expand and contract.	
			Valvular dysfunction	The development of problems with the heart's valves, affecting their ability to open and close properly.	
		Constitutional	Anorexia	Loss of appetite or a lack of interest in eating.	
			Fever	> 37.5 °C body temperature.	
			Lymphadenopathy	Swelling or enlargement of the lymph nodes.	
			Splenomegaly	Enlargement of the spleen.	
			Weight loss	> 5% unintentional body weight loss.	
		Gastrointestinal	Abdominal serositis or ascites	Lupus associated inflammation of the serous membranes in the abdomen, or the accumulation of fluid in the abdominal cavity.	
			Acute lupus cholecystitis	A sudden inflammation of the gallbladder due to lupus.	
			Acute lupus pancreatitis	A sudden inflammation of the pancreas caused by lupus.	
			Lupus enteritis/colitis	Inflammation of the intestines/colon due to lupus, causing gastrointestinal symptoms.	
			Lupus hepatitis	Liver inflammation caused by lupus, which can lead to liver dysfunction.	
			Intestinal pseudo-obstruction	A condition where the intestines do not move properly, mimicking a mechanical obstruction but without a physical blockage.	
			Malabsorption	Inability of the body to properly absorb nutrients from food due to lupus.	
			Protein losing enteropathy	Loss of protein through the gastrointestinal tract, which can lead to malnutrition and other complications.	
		Hematological	Active hemolysis	Raised bilirubin or raised reticulocyte count or reduced haptoglobulins or fragmented red blood cells or microspherocytes and positive Coombs’ test.	
			Hemoglobin	Test of hemoglobin concentration to detect whether patient have anemia, exclude dietary deficiency & GI blood loss	
			Lymphocyte count	Counts of lymphocytes to detect whether patient have lymphocytopaenia, exclude drug-induced cause.	
			Neutrophils	Counts of neutrophils to detect whether patient have neutropenia, exclude drug-induced cause.	
			Thrombocytopenia	Counts of platelets to detect whether patient have thrombocytopaenia, exclude drug-induced cause.	
			Thrombotic thrombocytopaenic purpura	Thrombotic thrombocytopaenic purpura with clinical syndrome of micro-angiopathic hemolytic anemia and thrombocytopenia in absence of any other identifiable cause.	
		Mucocutaneous	Alopecia	Hair loss.	
			Angioedema	Swelling beneath the skin or mucous membranes.	
			Bullous lupus	Inflammation of the fat layer beneath the skin.	
			Cutaneous vasculitis	Inflammation of the blood vessels in the skin that can lead to blood clots, resulting in skin lesions or ulcers.	
			Mucosal ulcers	Formation of ulcers or open sores on the mucous membranes.	
			Myositis	Inflammation of the muscles, which can cause pain, weakness, and stiffness.	
			Nodular vasculitis	Small, linear bleeding under the nails, resembling splinters. Tissue death (infarction) in the fingers or toes due to blockage of blood flow, or the presence of nodules caused by inflammation of the blood vessels.	
			Periungual erythema	Redness around the nails, often associated with cold exposure, which can lead to painful swelling and sometimes ulceration.	
			Skin eruption	Rash or outbreak on the skin.	
		Musculoskeletal	Arthritis	Inflammation of one or more joints, typically causing pain, swelling, and reduced range of motion.	
			Myalgia	Muscle or joint pain or inflammation.	
			Tendonitis	Inflammation of a tendon (tendonitis) or its sheath (tenosynovitis), leading to pain and difficulty moving the affected area.	
		Neuropsychiatric	Aseptic meningitis	Inflammation of meninges surrounding the brain and spinal cord without a bacterial infection.	
			Autonomic disorder	A dysfunction of the autonomic nervous system, which controls involuntary bodily functions like heart rate and digestion.	
			Cerebellar ataxia	A condition characterized by problems with coordination and balance due to damage to the cerebellum.	
			Cerebral vasculitis	Inflammation of the blood vessels in the brain.	
			Cerebrovascular stroke	Conditions affecting the blood vessels supplying the brain, such as stroke or transient ischemic attack.	
			Cognitive dysfunction	A decline in cognitive abilities, such as memory, attention, and problem-solving.	
			Delirium	Sudden onset of confusion.	
			Demyelinating syndrome	A condition where the protective covering (myelin) of nerve fibers is damaged, affecting nerve function.	
			Headache	A severe, continuous headache that is a symptom of systemic lupus erythematosus or a headache caused by increased pressure within the skull, often due to a buildup of cerebrospinal fluid.	
			Movement disorder	A group of neurological disorders that impair the body's ability to move.	
			Myelitis	Damage to the spinal cord, which can cause weakness, pain, and other neurological symptoms.	
			Peripheral neuropathy	A form of Guillain-Barré syndrome, where there is rapid onset of inflammation and damage to multiple peripheral nerves. Damage or dysfunction affecting one or more of the 12 pairs of cranial nerves, leading to a range of symptoms affecting many parts of the body.	
			Plexopathy	Damage to a nerve plexus, often causing pain and weakness.	
			Psychosis	A mental state characterized by a disconnection from reality, often involving hallucinations or delusions.	
			Seizures	A condition characterized by recurrent seizures, which are sudden, uncontrolled electrical disturbances in the brain. Or, a life-threatening medical condition characterized by continuous or near-continuous seizures.	
		Optic	Anterior ischemic optic neuropathy	A condition where the blood supply to the front part of the optic nerve is reduced, leading to sudden vision loss.	
			Anterior uveitis	Inflammation of the front part of the uvea, including the iris and ciliary body.	
			Episcleritis	Inflammation of the thin layer of tissue that covers the sclera.	
			Isolated cotton-wool spots (cytoid bodies)	Small, fluffy patches on the retina, which can be a sign of retinal or vascular disease.	
			Keratitis	Inflammation of the cornea, the clear front surface of the eye, which can impair vision.	
			Optic neuritis	Inflammation of the optic nerve, which can cause vision loss.	
			Orbital inflammation/myositis/proptosis	Inflammation of the tissues around the eye, muscle inflammation, or bulging of the eye.	
			Retinal vasculitis or hemorrhages	Inflammation of the back part of the uvea, including the retina and its blood vessels. Blockage of the blood vessels in the retina or choroid, which can lead to vision loss.	
			Scleritis	Inflammation of the sclera, which can be painful and potentially serious.	
		Renal	Accelerated renal hypertension	Blood pressure rising to > 170/110 mmHg within 1 month with grade 3 or 4 Keith-Wagener-Barker retinal changes (flame-shaped hemorrhages or cotton-wool spots or papilloedema).	
			Active nephritis	Inflammation of kidney with 3 months, conform to WHO classification (1995): (any one) Class III – (a) or (b) subtypes, Class IV – (a), (b) or (c) subtypes, Class V – (a), (b), (c) or (d) subtypes, vasculitis; or ISN/RPS classification (2003): (any one) Class III – (A) or (A/C) subtypes, Class IV – (A) or (A/C) subtypes, Class V, vasculitis. Glomerular sclerosis without inflammation not included.	
			Active urinary sediment	Pyuria (> 5 white blood cells/high power field or > 10 white blood cells/mm^3^) or hematuria (> 5 red blood cells/high power field or > 10 red blood cells/mm^3^) or red cell casts or white cell casts, exclude other causes (especially infection, vaginal bleed, calculi).	
			Creatinine	A waste product that is measured in blood tests to evaluate kidney function.	
			Diastolic blood pressure	Test of the pressure in the arteries when the heart rests between beats.	
			Glomerular filtration rate	The amount of blood filtered by the kidneys each minute and increas of which indicate deteriorate kidney function.	
			Lupus nephritis	Inflammation of the kidneys that can lead to kidney damage.	
			Nephrotic syndrome	A kidney disorder characterized with heavy proteinuria (>3.5 g/day or protein-creatinine ratio >350 mg/mmol or albumin-creatinine ratio >350 mg/mmol) and hypoalbuminaemia and edema.	
			Systolic blood pressure	Test of the pressure in the arteries when the heart beats and is pushing blood through the body.	
			Urine albumin-creatinine ratio	A test that compares the amount of albumin (a type of protein) to creatinine in urine.	
			Urine dipstick protein	A test that measures the presence of protein in urine.	
			Urine protein-creatinine ratio	A test measures the ratio of total protein to creatinine in urine.	
			Urine protein	The total amount of protein in urine collected over 24 h, used to assess kidney function.	
SELENA-SLEDAI	Activity	Cardiopulmonary	Pericarditis	Classic and severe pericardial pain or rub or effusion, or electrocardiogram confirmation.	^85^
			Pleurisy	Classic and severe pleuritic chest pain or pleural rub or effusion or new pleural thickening due to lupus.	
		Constitutional	Fever	>38 °C. Exclude infectious cause.	
		Hematological	Leukopenia	<3000 white blood cells/mm.^3^ Exclude drug causes.	
			Thrombocytopenia	<100,000 platelets /mm.^3^	
		Immunologic	Increased DNA binding	>25% binding by Farr assay or above normal range for testing laboratory.	
			Low complement	Decrease in 50% hemolytic complement, C3 or C4 below the lower limit of normal for testing laboratory.	
		Mucocutaneous	Alopecia	Ongoing abnormal, patchy or diffuse loss of hair due to active lupus.	
			Mucosal ulcers	Ongoing oral or nasal ulcerations due to active lupus.	
			New rash	Ongoing inflammatory lupus rash.	
		Musculoskeletal	Arthritis	More than 2 joints with pain and signs of inflammation (i.e., tenderness, swelling or effusion).	
			Myositis	Proximal muscle aching/weakness, associated with elevated creatine phosphokinase/aldolase or electromyogram changes or a biopsy showing myositis.	
		Neuropsychiatric	Cerebrovascular accident	New onset of cerebrovascular accident(s). Exclude arteriosclerosis or hypertensive causes.	
			Cranial nerve disorder	New onset of sensory or motor neuropathy involving cranial nerves. Include vertigo due to lupus.	
			Lupus headache	Severe persistent headache: may be migrainous, but must be nonresponsive to narcotic analgesia.	
			Organic brain syndrome	Altered mental function with impaired orientation, memory or other intellectual function, with rapid onset and fluctuating clinical features. Include clouding of consciousness with reduced capacity to focus, and inability to sustain attention to environment, plus at least 2 of the following: perceptual disturbance, incoherent speech, insomnia or daytime drowsiness, or increased or decreased psychomotor activity. Exclude metabolic, infectious or drug causes.	
			Psychosis	Altered ability to function in normal activity due to severe disturbance in the perception of reality. Include hallucinations, incoherence, marked loose associations, impoverished thought content, marked illogical thinking, bizarre, disorganized or catatonic behavior. Exclude uremia and drug causes.	
			Seizures	Recent onset (last 10 days). Exclude metabolic, infectious or drug cause, or seizure due to past irreversible central nervous system damage.	
		Optic	Visual disturbance	Retinal and eye changes of SLE. Include cytoid bodies, retinal hemorrhages, serous exudate or hemorrhages in the choroid, optic neuritis, scleritis or episcleritis. Exclude hypertension, infection or drug causes.	
		Renal	Hematuria	>5 red blood cells/high power field. Exclude stone, infection or other cause.	
			Proteinuria	New onset or recent increase of more than 0.5 g/24 h.	
			Pyuria	>5 white blood cells/high power field. Exclude infection.	
			Urinary casts	Heme-granular or red blood cell casts.	
		Vascular	Carditis	Inflammation of the heart.	
SLAM	Activity	Cardiopulmonary	Pleuritis	Inflammation of the pleural, may lead to fluid around the lungs, shortment of breath at rest or with exercise and decreased breath sounds and dull lower or even middle lobes.	^94^
			Pneumonitis	Inflammation of the lungs. Evidenced by X-ray, computed tomography, magnetic resonance imaging or ultra sound. May lead to shortness of breath with exercise or even at rest.	
			Vasculitis	Ulceration, gangrene, tender finger nodules, periungual infarction, splinter hemorrhages, or biopsy or angiogram proof of vasculitis.	
		Constitutional	Fatigue	May lead to functional limitation.	
			Fever	Body temperature higher than normal range.	
			Hepatomegaly and/or splenomegaly	Enlargement of liver and/or spleen which may be palpable with or without inspiration.	
			Lymphadenopathy	Swollen lymph nodes in various parts of the body, especially in cervical, axillary, epitrochlear area.	
			Weight loss	Unintentional weight loss.	
		Gastrointestinal	Abdominal pain	Pain in the abdominal area caused by serositis, pancreatitis, ischemic bowel, etc.	
			Hepatomegaly and/or splenomegaly	Enlargement of liver and/or spleen which may be palpable with or without inspiration.	
		Hematological	Erythrocyte sedimentation rate	Detect of erythrocyte sedimentation rate, increasing of which may hint inflammation.	
			Hematocrit	The proportion of space in the blood filled with red blood cells, decrease of which may hint anemia.	
			Lymphocyte count	Counts of lymphocytes to detect whether patient have lymphocytopaenia.	
			Thrombocytopenia	Counts of platelets to detect whether patient have thrombocytopaenia.	
			White blood cell count	Counts of white blood cells to detect whether patient have leukocytopaenia.	
		Mucocutaneous	Alopecia	Loss of hair.	
			Bullous lesions	Severe vacuolar alteration at the dermal-epidermal junction leading to development of blisters.	
			Discoid lupus	Erythematous-violaceous cutaneous lesions with secondary changes of atrophic scarring, dyspigmentation. Lesions have a preference for the head and neck, especially the conchal bowl, but may be found in nearly any location.	
			Erythematous	Red rash.	
			Lupus profundus	Intense inflammation in the fat leads to indurated plaques that can evolve into disfiguring, depressed areas.	
			Maculopapular rash	Raised skin rash.	
			Malar rash	Fixed, flat or raised erythema (superficial reddening of the skin) over the malar eminences, but tends to spare the nasolabial folds.	
			Oral ulcers	Oral or nasopharyngeal ulceration, usually painless and based on physician examination.	
			Periungual erythema	Redness around the nails, often associated with cold exposure, which can lead to painful swelling and sometimes ulceration.	
			Photosensitive rash	Skin rash that appears or worsens with sun exposure.	
		Musculoskeletal	Joint pain	Pain in the joints cause of synovitis and/or tenosynovitis.	
			Myalgia	Muscle pain or inflammation.	
		Neuropsychiatric	Cerebrovascular stroke	Stroke caused by mononeuritis multiplex, transient ischemic attack, reversible ischemic neurologic deficit, cough variant asthma, retinal vascular thrombosis.	
			Cortical dysfunction	Impaired function of the brain's outer layer.	
			Headache	Pain in the head, including types similar to migraines.	
			Seizures	A sudden, uncontrolled electrical disturbance in the brain.	
		Optic	Cytoid bodies	Cotton-wool spots seen in retinal exams, which may affect visual acuity.	
			Papillitis or pseudotumor cerebri	Inflammation of the optic nerve head or increased pressure in the skull without a tumor, which may affect visual acuity or even lead to field cut.	
			Retinal vasculitis or hemorrhages	Bleeding in the retina or choroid or inflammation of the outer layer of the eye, which may affect visual acuity.	
		Renal	Creatinine	Serum creatinine is a blood test that measures the level of creatinine in the blood, indicating kidney function. Creatinine clearance is a calculation based on creatinine levels in urine and blood, used to estimate how well the kidneys are filtering waste.	
			Hypertension	Blood pressure higher than normal.	
			Urine sediment	>5 red blood cells and/ or white blood cells/high-power field and/or red cell cast and/or >4+ proteinuria and/or > 3.5 g/L /24h urine protein	
		Vascular	Nail fold infarct	Tissue death at the base of the nail due to blocked blood flow. Or, redness around the nails.	
			Raynaud's disease	A condition causing color changes in fingers or toes due to reduced blood flow.	
			Vasculitis	Inflammation of blood vessels leading to presentations such as leucocytoclastic casculitis, urticaria, palpable purpura, livedo reticularis, ulcer or panniculitis.	
SLEDAI	Activity	Cardiopulmonary	Pleurisy	Pleuritic chest pain with pleural rub or effusion, or pleural thickening.	^95^
			Pericarditis	Pericardial pain with at least 1 of the following: rub, effusion, or electrocardiogram or echocardiogram confirmation.	
		Constitutional	Fever	>38°C. Exclude infectious cause.	
		Hematological	Leukopenia	<3,000 white blood cells/mm.^3^ Exclude drug causes.	
			Thrombocytopenia	<100,000 platelets/mm.^3^	
		Immunologic	Increased DNA binding	>25% binding by Farr assay or above normal range for testing laboratory.	
			Low complement	Decrease in 50% hemolytic complement, C3, or C4 below the lower limit of normal testing laboratory.	
		Mucocutaneous	Alopecia	New onset or recurrent abnormal, patchy or diffuse loss of hair.	
			Mucosal ulcers	New onset or recurrent Oral or nasal ulcerations.	
			New rash	New onset or recurrent of inflammatory type rash.	
		Musculoskeletal	Arthritis	More than 2 joints with pain and signs of inflammation (i.e., tenderness, swelling or effusion).	
			Myositis	Proximal muscle aching/weakness, associated with elevated creatine phosphokinase/aldolase or electromyogram changes or a biopsy showing myositis.	
		Neuropsychiatric	Cerebrovascular accident	New onset of cerebrovascular accident(s). Exclude arteriosclerosis.	
			Cranial nerve disorder	New onset of sensory or motor neuropathy involving cranial nerves.	
			Lupus headache	Severe, persistent headache, may be migrainous, but must be nonresponsive to narcotic analgesia.	
			Organic brain syndrome	Altered mental function with impaired orientation, memory, or other intellectual function, with rapid onset and fluctuating clinical features, include clouding of consciousness with reduced capacity to focus, and inability to sustain attention to environment, plus at least 2 of the following: perceptual disturbance, incoherent speech, insomnia or daytime drowsiness, or increased or decreased psychomotor activity. Exclude metabolic, infectious, or drug causes.	
			Psychosis	Altered ability to function in normal activity due to severe disturbance in the perception of reality, include hallucinations, incoherence, marked loose associations, impoverished thought content, marked illogical thinking, bizarre, disorganized, or catatonic behavior. Exclude uremia and drug causes.	
			Seizures	Recent onset, exclude metabolic, infectious or drug causes,	
		Optic	Visual disturbance	Retinal changes of SLE, include cytoid bodies, retinal hemorrhages, serous exudate or hemorrhages in the choroid, or optic neuritis. Exclude hypertension, infection, or drug causes.	
		Renal	Hematuria	>5 red blood cells/high power field. Exclude stone, infection or other cause.	
			Proteinuria	>0.5 g/24 h. New onset or recent increase of more than 0.5 g/24 h.	
			Pyuria	>5 white blood cells/high power field. Exclude infection.	
			Urinary casts	Heme-granular or red blood cell casts.	
		Vascular	Vasculitis	Ulceration, gangrene, tender finger nodules, periungual infarction, splinter hemorrhages, or biopsy or angiogram proof of vasculitis.	
SLEDAI-2K	Activity	Cardiopulmonary	Pericarditis	Pericardial pain with at least 1 of the following: rub, effusion, electrocardiogram or echocardiogram confirmation.	^91^
			Pleurisy	Pleuritic chest pain with pleural rub or effusion, or pleural thickening.	
		Constitutional	Fever	>38 °C. Exclude infectious cause.	
		Hematological	Leukopenia	<3000 white blood cells/mm.^3^ Exclude drug causes.	
			Thrombocytopenia	<100,000 platelets/mm.^3^ Exclude drug causes.	
		Immunologic	Increased DNA binding	Increased DNA binding above normal range for testing laboratory.	
			Low complement	Decrease in 50% hemolytic complement, C3, or C4 below the lower limit of normal far testing laboratory	
		Mucocutaneous	Alopecia	Abnormal, patchy or diffuse loss of hair.	
			Mucosal ulcers	Oral or nasal ulcerations.	
			New rash	Inflammatory-type rash.	
		Musculoskeletal	Arthritis	≥2 joints with pain and signs of inflammation (i.e., tenderness, swelling, or effusion).	
			Myositis	Proximal muscle aching/weakness associated with elevated CK/aldolase or electromyography changes or a biopsy showing myositis.	
		Neuropsychiatric	Cerebrovascular accident	New onset of cerebrovascular accident(s). Exclude arteriosclerosis.	
			Cranial nerve disorder	New onset of sensory or motor neuropathy involving cranial nerves.	
			Lupus headache	Severe, persistent headache, may be migrainous, but must be nonresponsive to narcotic analgesia.	
			Organic brain syndrome	Altered mental function with impaired orientation, memory, or other intellectual function, with rapid onset and fluctuating clinical features, inability to sustain attention to environment, and at least two of the following: perceptual disturbance, incoherent speech, insomnia or daytime drowsiness, and increased or decreased psychomotor activity. Exclude metabolic, infectious, or drug causes.	
			Psychosis	Altered ability to function in normal activity due to severe disturbance in the perception of reality, include hallucinations, incoherence, marked loose associations, impoverished thought content, marked illogical thinking, and bizarre, disorganized, or catatonic behavior. Exclude uremia and drug causes.	
			Seizures	Recent onset, exclude metabolic, infectious or drug causes,	
		Optic	Visual disturbance	Retinal changes of SLE, include cytoid bodies, retinal hemorrhages, serous exudates or hemorrhages in the choroid, or optic neuritis. Exclude hypertension, infection, or drug causes.	
		Renal	Hematuria	>5 red blood cells/high-power field. Exclude stone, infection, or other cause.	
			Proteinuria	>0.5 g/24 h	
			Pyuria	>5 white blood cells/high power field. Exclude infection.	
			Urinary casts	Heme-granular or red blood cell casts.	
		Vascular	Vasculitis	Ulceration, gangrene, tender finger nodules, periungual infarction, splinter hemorrhages or biopsy, and angiogram proof of vasculitis.	
SLEDAI 2000 Responder Index 50	Activity	Cardiopulmonary	Pericarditis	≥50% reduction in the pain severity as determined by patient on numerical scale of 1-10 and/or ≥50% reduction in the amount of fluid (on imaging) with no worsening in either.	^111^
			Pleurisy	≥50% reduction in the pain severity as determined by patient on numerical scale of 1-10 and/or ≥50% reduction in the amount of fluid (on imaging) with no worsening in either.	
		Constitutional	Fever	≥50% reduction in the degree of fever above normal.	
		Hematological	Leukopenia	≥50% increase in the level of white blood cells but <3,000/mm.^3^	
			Thrombocytopenia	≥50% increase in the level of platelets but <100,000 platelets/mm.^3^	
		Immunologic	Increased DNA binding	≥50% reduction in the level of anti-DNA antibodies.	
			Low complement	≥50% increase in the level of any complement or normalization of one of them without a drop in either.	
		Mucocutaneous	Alopecia	≥50% decrease of total scalp involved area for patchy alopecic lesion or ≥50% reduction in the diffuse alopecia as determined by patient on numerical scale of 1-10, and/or activity of the most active alopecic lesions with no worsening in cither. Activity of the alopecic lesion should be determined by the color of the most active lesion: 0-absent, 1-pink (faint erythema), 2-red, 3-dark red/purple/violaceous/crusted/hemorrhagic. ≥50% decrease in the activity of the lesion is defined by decreasing by 2 grades.	
			Mucosal ulcers	≥50% decrease in the number of ulcers at this visit.	
			New rash	≥50% decrease of involved body surface area and/or activity of most active lesion with no worsening in either. Activity of the lesion should be determined by the color of the lesions: 0-absent, 1-pink (faint erythema), 2-red, 3-dark red/purple/violaceous/crusted/hemorrhagic. ≥50% decrease in the activity of the lesion is defined by decreasing by 2 grades. Dyspigmentation, scarring and atrophy are not active lesions.	
		Musculoskeletal	Arthritis	≥50% reduction in the number of joints with pain and signs of inflammation (i.e., tenderness, swelling or effusion).	
			Myositis	≥50% increase in muscles power judged by physician or increase by or 1 grade upon a scale of zero to five or ≥50% decrease in the level of creatinine phosphokinase/aldolase level comparing to previous visit with no worsening in either.	
		Neuropsychiatric	Cranial nerve disorder	≥50% recovery of motor or sensory function in affected nerve within 1 month from the event on the basis of decrease in lupus disease activity or ≥50% decrease of the severity of pain within 1 month from the event on the basis of decrease in lupus disease activity as determined by patient on numerical scale of 1–10 if applicable with no worsening in either.	
			Lupus headache	≥50% decrease of the severity of pain as determined by patient on numerical scale of 1–10.	
			Organic brain syndrome	≥50% improvement of the psychotic manifestations judged by physician.	
			Psychosis	≥50% improvement of the psychotic manifestations judged by physician.	
			Cerebrovascular accident	≥50% recovery of motor or sensory function related to cerebrovascular accident within 1 month from the event on the basis of decrease in lupus disease activity as determined by physician without worsening in either.	
			Seizures	≥50 % reduction in frequency of baseline seizure days/month.	
		Optic	Visual disturbance	≥50% improvement of the psychotic manifestations judged by physician.	
		Renal	Hematuria	≥50% decrease in the number of red blood cell /high power field at this visit	
			Proteinuria	≥50% decrease in the range of proteinuria.	
			Pyuria	≥50% decrease in the number of white blood cells/ high power field.	
			Urinary casts	≥50% decrease in the total number of heme-granular and red blood cell casts.	
		Vascular	Vasculitis	≥50% improvement of the vasculitis lesions present with no new lesion or worsening in either, ≥50% improvement for ulceration or gangrene is defined as ≥50% decrease in the body surface area, for periungual infarction, splinter hemorrhages or tender finger nodules a ≥50% improvement is defined as ≥50% decrease in the total number of involved digits with periungual infarction, splinter hemorrhages and tender finger nodules. Multiple lesions in a single digit, count only one.	
SLE-DAS	Activity	Cardiopulmonary	Diffuse alveolar hemorrhage	A critical and potentially life-threatening condition characterized by bleeding into the alveolar spaces of the lungs.	^98^
			Libman-Sacks endocarditis	An inflammation of the inner lining of the heart chambers and heart valves, characterized by the formation of vegetations.	
			Myocarditis	Inflammation of the heart muscle, which can affect its function and cause various symptoms.	
			Pleuritis or pericarditis	Including sterile peritonitis in addition to pleurisy and pericarditis.	
			Pneumonitis	Inflammation of the alveoli and the tissue surrounding them, causing difficulty breathing.	
			Pulmonary hypertension	High blood pressure that affects the arteries in the lungs and the right side of the heart.	
			Shrinking lung	Lungs become smaller and stiffer, reducing their ability to expand and contract.	
			Valvular dysfunction	Malfunction of heart's valves, affecting their ability to open and close properly.	
		Hematological	Hemolytic anemia	Anemia with positive direct Coombs test, increased serum lactate dehydrogenase and low serum haptoglobin.	
			Leucopenia	Leukocyte count (10^9^/L), below 3 × 10^9^/L white blood cells.	
			Thrombocytopenia	Platelet count (10^9^/L), below 100 × 10^9^/L platelets.	
		Immunologic	Hypocomplementaemia	Decrease in C3 or C4 below the lower limit of normal for testing laboratory.	
			Positive anti-nuclear autoantibody	Increase in DNA binding above the upper limit of normal for testing laboratory.	
		Mucocutaneous	Alopecia	Abnormal, patchy or diffuse loss of hair.	
			Generalized skin rash	Acute, subacute and chronic cutaneous lupus rashes included in the SLICC classification criteria, above and below the neck.	
			Localized skin rash	Acute, subacute and chronic cutaneous lupus rashes included in the SLICC classification criteria, only above the neck.	
			Mucocutaneous vasculitis	Any mucocutaneous vasculitis and chilblain lupus.	
			Oral ulcers	Oral or nasal ulcerations.	
		Musculoskeletal	Arthritis	Number of swollen joints in 28-joint count.	
			Myositis	Proximal muscle aching/weakness with elevated CK/aldolase or electromyogram changes or a biopsy showing myositis.	
		Neuropsychiatric	Aseptic meningitis	Inflammation of meninges surrounding the brain and spinal cord without a bacterial infection.	
			Cerebrovascular accident	A sudden event in the brain caused by a blockage or rupture of a blood vessel, leading to a lack of blood flow and oxygen to brain cells.	
			Delirium	A sudden and severe disturbance of consciousness and cognitive function.	
			Headache	Headaches that occur in individuals with lupus, which can range from mild to severe and may be related to the inflammation caused by the disease.	
			Myelitis	Any disease or condition that affects the spinal cord, leading to various neurological symptoms depending on the location and severity of the damage.	
			Organic brain syndrome	A group of symptoms caused by a physical disorder in the brain, such as memory loss, mood changes, and confusion.	
			Peripheral neuropathy	Damage to the peripheral nerves, which can cause pain, numbness, tingling, or weakness in the extremities.	
			Psychosis	Delusions and/or hallucinations without insight and absence of delirium.	
			Seizures	Primary generalised seizure or partial/focal seizure, with independent description by a reliable witness. If electroencephalography is performed, abnormalities must be present.	
		Optic	Proteinuria	Urinary protein-creatinine ratio (mg/g) or 24 h urinary protein (mg/24 h), above 500 mg/g and 500 mg/24 h, respectively.	
		Renal	Retinal changes	SLE caused inflammation in the retina, leading to changes that can affect vision.	
		Vascular	Systemic vasculitis	Systemic vasculitis involving large and medium-sized vessels and lupus enteritis.	
SLICC/ACR SDI index	Activity	Cardiopulmonary	Angina or coronary artery bypass	Angina refers to chest pain or discomfort due to reduced blood flow to the heart muscle. Coronary artery bypass refers to urgery to reroute blood around blocked or narrowed coronary arteries.	^113^
			Cerebrovascular stroke	Having medical history of stroke at any point.	
			Chronic peritonitis	Ongoing inflammation of the peritoneum.	
			Myocardial infarction	Damage to the heart muscle due to blocked blood flow, often causing chest pain.	
			Pleural fibrosis	Scarring of the tissue lining the lungs.	
			Pleuritis or pericarditis	Inflammation of the sac around the heart for at least 6 months, or surgery to remove it.	
			Pulmonary fibrosis	Scarring of lung tissue.	
			Pulmonary hypertension	High blood pressure that affects the arteries in the lungs and the right side of the heart.	
			Pulmonary infarction	Tissue death in the lungs due to blocked blood flow.	
			Shrinking lung	Lungs become smaller and stiffer, reducing their ability to expand and contract.	
			Valvular dysfunction	Problems with the heart's valves, which can cause a heart murmur or difficulty in blood flow.	
		Comorbid	Diabetes	A condition characterized by high blood sugar levels.	
			Malignancy	Cancer.	
			Premature gonadal failure	Early failure of the reproductive glands.	
		Gastrointestinal	Infarction or resection of bowel below duodenum, spleen, liver, or gall bladder ever, for cause any	Tissue death or surgical removal of parts of the bowel below duodenum, spleen, liver, or gall bladder due to various causes.	
			Mesenteric insufficiency	Reduced blood flow to the intestines, which can impair their function and cause abdominal pain and/or gastrointestinal bleeding.	
			Stricture or upper gastrointestinal tract surgery ever	Narrowing of the digestive tract or surgeries performed on it.	
		Mucocutaneous	Alopecia	Permanent hair loss due to scarring.	
			Extensive scarring or panniculus other than scalp and pulp space	Large areas of scarring or fat atrophy.	
			Skin ulceration	Sores on the skin lasting more than 6 months, not caused by blood clots.	
		Musculoskeletal	Avascular necrosis	Death of bone tissue due to lack of blood supply.	
			Claudication	Leg pain when walking due to poor blood flow, lasting for 6 months.	
			Deforming or erosive arthritis	Joint damage causing deformity or erosion.	
			Muscle atrophy or weakness	Wasting or weakness of muscles.	
			Osteomyelitis	Bone infection.	
			Osteoporosis with fracture or vertebral collapse	Thinning of bones with fractures or spinal collapses.	
		Neuropsychiatric	Myelitis	Inflammation across the spinal cord that can cause pain, weakness, and sensory loss.	
			Cranial neuropathy	Cranial neuropathy refers to damage or impairment of the cranial nerves, which can result in facial weakness, vision changes, or sensory disturbances in the head.	
			Peripheral neuropathy	Peripheral neuropathy refers to damage to the nerves outside the central nervous system, leading to symptoms like numbness, tingling, and muscle weakness in the limbs.	
			Psychosis	Severe disturbance in the perception of reality, characterized by delusions and/or hallucinations. Symptoms like memory deficit, difficulty with calculation, poor concentration, difficulty in spoken or written language, impaired performance level.	
			Seizures	Primary generalised seizure or partial/focal seizure, with independent description by a reliable witness. If electroencephalography is performed, abnormalities must be present. Requiring therapy for 6 months.	
		Optic	Cataract	Clouding of the eye's natural lens, which can cause vision loss and often requires surgery to treat.	
			Retinal change or optic atrophy	Retinal change refers to any alteration in the appearance or function of the retina. Optic atrophy is the degeneration of the optic nerve fibers, often leading to vision loss, and is identifiable through an examination of the fundus.	
		Renal	Low glomerular filtration rate	>50% decrease glomerular filtration rate.	
			Proteinuria	Urine protein ≥3.5 g/24 h.	
			Renal disorder	Severe, irreversible kidney disease.	
		Vascular	Minor tissue loss	Small area of tissue damage, such as in the fingertip due to poor blood supply.	
			Significant tissue loss	Major loss of tissue, such as a part of a finger or an entire limb due to poor blood supply.	
			Venous thrombosis	Formation of blood clots within veins and accompanied with accumulation of fluid in the tissues or formation of ulcers.	
			Venous stasis	Blood clot in a vein, with symptoms like swelling and skin ulceration.	
BICLA response	Activity	NA	NA	Reduction in any moderate-to-severe baseline disease activity and no worsening in any of nine organ systems in the BILAG index, no worsening on the SLEDAI, no increase of 0.3 points or more in the score on the Physician Global Assessment of disease activity (on a scale from 0 [no disease activity] to 3 [severe disease]), no discontinuation of the trial intervention, and no use of medications restricted by the protocol.	^104^
LLDAS	Activity	NA	NA	(1)S LEDAI-2K ≤4, with no activity in major organ systems (renal, central nervous system, cardiopulmonary, vasculitis, fever) and no hemolytic anemia or gastrointestinal activity; (2) No new lupus disease activity compared with the previous assessment; (3) SELENA-SLEDAI physician global assessment (scale 0–3) ≤1; (4) A current prednisolone (or equivalent) dose ≤ 7.5 mg daily; (5) Well tolerated standard maintenance doses of immunosuppressive drugs and approved biological agents.	^119^
SRI response	Activity	NA	NA	(1) >4-point reduction in SELENA-SLEDAI; (2) No new BILAG A or no more than 1 new BILAG B domain score, (3) No deterioration from baseline in the physician's global assessment by >0.3 points.	^112^

*ACR* American College of Rheumatology, *ANA* antinuclear antibody, *BICLA* British Isles Lupus Assessment Group-based Composite Lupus Assessment, *BILAG* British Isles Lupus Assessment Group, *LLDAS* Lupus Low Disease Activity State, *SELENA-SLEDAI* Safety of Estrogens in Lupus Erythematosus National Assessment Version of the Systemic Lupus Erythematosus Disease Activity Index, *SLAM* Systemic Lupus Activity Measure, *SLE-DAS* Systemic Lupus Erythematosus Disease Activity Score, *SLICC/ACR* SDI index Systemic Lupus International Collaborating Clinics/American College of Rheumatology Damage Index for Systemic Lupus Erythematosus, *SLICC* Systemic Lupus International Collaborating Clinics, *SRI* Systemic Lupus Erythematosus Responder Index

The ACR criteria have been considered to be more feasible for classifying advanced SLE patients. This is because that the 1997-version requires the presence of no less than four items and the 2019-version requires even more indexes, as well as the fact that the symptoms accrue as the disease progresses.^87,88,90^

It is worth noting that the ACR criteria include the most prevalent manifestations but not all. For instance, the mucocutaneous manifestations included in the ACR criteria focus on discoid lupus and oral ulcers that do not cover other skin symptoms such as subacute cutaneous lupus, psoriasiform, and other forms of chronic cutaneous lupus; clinical syndromes from the neurological system is poorly represented in both the 1997 and 2019 versions of the ACR criteria that lack other important manifestations such as organic brain syndrome and cerebrovascular accident.^91^ Other SLE classification criteria also exist such as the Systemic Lupus International Collaborating Clinics (SLICC) criterion that overcomes issues faced by the ACR criterion such as the lack of cutaneous and neuropsychiatric manifestations^92^ (Table 1). However, the specificity of the SLICC criterion drops from 93% to 84% despite its comparable sensitivity (i.e., 97%) with the 2019-version ACR criteria (i.e., 96%) due to the large spectrum of SLE manifestations it includes.^87,92^ Also, the SLICC classification criteria did not make substantial improvement in diagnosing patients with early disease onset as compared with the ACR criterion. Thus, unless more clever criteria with both increased sensitivity and specificity could be developed, the 2019-version ACR criterion may remain prevalent for SLE diagnosis. However, to enable accurate diagnosis of SLE with only a few indexes is challenging due to the extreme heterogeneous nature of this disease regarding its diversified clinical manifestations. One possibility would be to use molecular markers that requires in-depth understandings on SLE pathogenesis and identification of the leading signaling axis or panel of molecules marking the initiation and/or progression of SLE. Specifically, stratifying the pathogenic process of SLE into vital stages and identifying markers characterizing each phase may clearly mark the disease cause and activity of an individual on diagnosis. This may not only aid in the therapeutic design for precision medicine despite the heterogeneity nature of this disease, but also enable early diagnosis as markers are grouped by the stages following the line of disease initiation and progression.

### Disease activity

Assessment of SLE activity conveys significant clinical values as it is a prognostic factor associated with mortality.^93^ However, accurate SLE activity measure is challenging due to the multifaceted clinical manifestations SLE possess and its extreme variation features over time.

Multiple indexes have been developed to assess the activity of SLE from multiple dimensions. The Systemic Lupus Activity Measure (SLAM) is a scale developed to assess the disease activity of SLE, which comprises items across 11 organ systems but does not require immunological test results and can be scored based solely on the physician’s clinical examination, making it applicable in areas where laboratory testing is limited. SLAM has a relatively satisfying high sensitivity and is currently widely used around the world.^94^ The SLEDAI^95^ together with its updated versions^85,91,96^ have been used to describe the overall burden of SLE. The Adjusted Mean SLEDAI-2000 (AMS) has been developed to measure the disease activity over time.^97^ However, SLEDAI and it updated versions have limitations in detecting clinically meaningful changes in the disease activity, as only complete remission but not partial improvement of the disease status can be captured using SLEDAI. Thus, SLE disease activity score (SLE-DAS) has been established accordingly to overcome such obstacles, which has displayed a desirable sensitivity for assessing alterations in disease activity.^98^ The British Isles Lupus Assessment Group (BILAG) criteria and revisions^99–101^ are organ-specific indices assessing the partial improvement of SLE activity that can be used alone or as part of a composite index such as the BILAG-based Composite Lupus Assessment (BICLA).^102^ BICLA is integrated from BILAG, SLEDAI and Physician Global Assessment (PGA),^103,104^ which can comprehensively evaluate the benefits of an individual patient from a particular therapeutic towards an efficient utilization of the medical resources,^105^ and thus has been adopted by several clinical trials.^106–110^ Similarly, the SLEDAI-2000 Responder Index-50^111^ and composite indices such as the SLE Responder Index (SRI)^112^ are also available for assessing partial SLE improvement. The SLICC ACR Damage Index (SDI) has been developed to measure the accumulated organ damage ever since the disease onset,^113^ which has been shown to be a reliable^114^ independent outcome measure^115^ as well as a predictive index on future damage accrual and mortality.^116^ In addition, lupus low disease activity (LLDAS) has been used to describe prolonged disease remission or a serologically active (i.e., high anti-dsDNA antibody or low levels of complements) but clinically quiescent period among SLE patients.^117^ Therapeutically, patients during the LLDAS phase do not need specific treatment but require close surveillance^118,119^ (Table 1).

### Disease comorbidities

As a direct result of SLE or a consequence of SLE medications such as glucocorticoids, SLE patients are at a high risk of developing several comorbidities. Thus, surveillance using measurements of each type of comorbidity should be adopted to prevent SLE patients from developing these diseases, which is crucial to their early identification and the prevention/intervention of these SLE-associated disorders among these patients.

Primary SLE comorbidities include cancers such as hematological malignancies, cardiovascular disorders such as atherosclerosis, bone diseases such as osteonecrosis, and neuropsychiatric symptoms such as cognitive dysfunction. In particular, SLE is associated with an increased risk of developing cancers, especially breast cancers, cervical cancers, hematological cancers, and lung cancers,^120^ with cancer screening being recommended for SLE carriers by the EULAR.^9^ SLE has been considered as a risk factor for atherosclerosis and been incorporated into the American Heart Association guidelines for cerebrovascular disease prevention among women.^10^ In addition, coronary artery disease was documented in 6-11% SLE patients, and subclinical carotid plaque was reported in 30-50% SLE carriers.^121^ SLE patients are at a high risk of developing osteonecrosis and osteopenia. Specifically, the incidences of osteoporosis and osteopenia occur in 1.4-68% and 25-74% SLE patients, respectively.^122^ Besides, SLE-related factors such as disease activity and medication use have been reported to be risky for developing low bone mineral density.^122^ Cognitive impairment occurs in up to 88% neuropsychiatric SLE^123^ that requires early diagnosis and appropriate interventions to prevent its long-term damage accumulation. Thus, there is an urgent need of standardized metrics for identifying cognitive impairment that, however, is lacking.^124^

## Risk factors of SLE

Risk factors of SLE can be classified into intrinsic and extrinsic levels, with intrinsic factors being further grouped into those occurring at the genetic, epigenetic and hormonal levels, and extrinsic factors classifiable into environmental factors, habits, physiological factors and phycological factors. Before going into details of each type of SLE risk factors, it is worth to note that factors predisposing the risk of developing SLE is multifactorial that can not be explained solely by information from any of these layers, and risk factors from multiple levels can synergize to predispose the onset and severity of SLE. As examples, increased risks of developing SLE have been observed when genetic risk factors interact with smoking,^125^ and when the risk allele of the gene encoding chymotrypsin-like elastase family A member 1 (CTYP24A1) is coupled with insufficient vitamin D supply.^126^

While we focus more on intrinsic factors in this section and go into details of those at the genetic, epigenetic and hormonal levels to gain insights for intrinsic SLE predisposition, we emphasize extrinsic factors in the following section to identify preventive approaches for practical advice.

### Genetic factors predisposing SLE

SLE is an autoimmune disease with a strong genetic disposition. Approximately 5-12% of people having one of his/her first-degree relatives carrying SLE will develop this disease in their lifetime.^127^ A series of landmark familial linkage studies and genome-wide association studies (GWAS) in SLE have greatly advanced our understanding regarding the genetic basis of SLE.^128–130^ Currently, more than 100 SLE susceptibility loci have been consecutively identified (mostly from the European and Asian populations), which can explain up to 30% of SLE inheritability.^131–134^

Being a multigenic disease, several weighted genetic risk scores (GRS) have been established to assess the cumulative genetic susceptibility of an individual to SLE,^125^ with a higher GRS being associated with an earlier SLE onset and a higher disease activity.^135^ Male SLE patients, in general, have higher GRS than female carriers, implicating that the genetic factors play more dominant roles among males than females in predisposing SLE susceptibility.^136^

Genetic factors predisposing the incidence of SLE include both high-risk rare mutations and high-frequency polymorphisms (SNPs) that aggregate to collectively enhance the susceptibility of an individual to SLE. Of note, though each conveying a small effect on SLE risk by itself, low-frequency SNPs, once aggregated in a sufficient amount, may deliver substantial impact on SLE susceptibility.^137^ Genetic alleles so far identified are largely distributed in genes participating in IFN-relayed signal transduction, genes encoding components of the MHC region and altering the threshold for activating T/B lymphocytes, and genes encoding elements of the complement system such as C2, C4, C1q that impair the clearance of cellular debris.^129,137–139^ Genetic factors predisposing SLE susceptibility can be classified into three categories according to the molecular mechanisms they may participate in, i.e., stimulating the immune system, skewing immune regulatory signals, and impairing the debris clearance machinery.

#### Genetic factors associated with stimulated immune response

A large number of SLE-associated SNPs have been mapped to genes encoding proteins regulating or in response to type I IFNs such as genetic variants of IFN regulatory factor (IRF) 5 and IRF7.^140^ These SLE-associated genes are known as the ‘IFN signature’ actively participating in the innate immune response, and SLE patients possessing high levels of IFNα are inclined to manifest more severe disease syndromes.^141^ Mechanically, type I IFNs are produced in response to foreign material invasion for promoted maturation of DCs and production of proinflammatory cytokines, leading to, e.g., stimulated Th1 polarization and B cell activation. *IRF5*, being one member of the IFN signature with critical roles in regulating type I IFN-responsive genes, conveys a modest contribution to SLE risk (with the odds ratio being 1.5) and is considered to be the most strongly SLE-associated gene outside of MHC.^142^ As one example of SNPs of this kind, rs12537284 was identified through GWAS followed by meta-analysis and candidate gene investigations.^143^ STAT4 has recognized as a susceptibility gene of SLE that carries an additive value with IRF5 for increased risk of developing SLE.^144^ Accordingly, SNP risk variants rs3821236, rs3024866, rs7574865, being associated with high levels of *STAT4* expression, conferred increased sensitivity to IFNα signaling in the peripheral blood mononuclear cells (PBMCs) of SLE patients and displayed earlier disease onset and more severe disease syndromes.^145^ Besides *IRF5*, SNPs associated with genes encoding other type I IFNs such as rs4963128 of *IRF7*^146^ and rs116440334 of *IRF8*^147^ have also been implicated in IFN pathways. Several other genes have also been identified capable of influencing IFNα signaling and the innate immune response. These include, e.g., *IRAK1* (encoding IL1 receptor-associated kinase 1) that can be used to explain the female-predominance feature of SLE,^148^ and *OPN* (encoding osteopontin) that is associated with early SLE onset.^149^

In addition, SNPs residing in the genetic elements of microRNA (miRNA) critical for relaying IFN signals have been characterized.^150^ For instance, rs57095329, located in the promoter region of miRNA-146a, has been found to be highly associated with SLE susceptibility.^150^ Specifically, individuals carrying the risky G allele exhibited significantly reduced level of miRNA-146a than those carrying the protective C allele; and this may be attributed to the altered binding affinity of the transcription factor (TF) ETS proto-oncogene 1 (Ets-1) to the promoter region of miRNA-146a as a result of this genetic polymorphism.^150^

#### Genetic factors associated with immune signal relay

Another important portfolio of genetic risk factors predisposing SLE development are located in genes associated with the MHC especially human leukocyte antigen (*HLA)-DRB1* in the MHC class II region.^151^ HLA molecules play vital roles in auto-antibody production, as risk residues associated with the production of characteristic auto-antibodies (i.e., DRB1 residues 11, 13, 30) being located in the peptide-binding groove of *HLA-DRB1*.^152^ Furthermore, *HLA-DRB1* and *HLA-DQB1* form the most significant haplotype, and seven residues (i.e., HLA-DRB1 residues 13, 11, 37, HLA-DQB1 residue 37, HLA-DPB1 residue 35, HLA-A residue 70, and HLA-B residue 9) collectively increase the explainable heritability of SLE due to HLA to 2.6%.^152^ Many other genes with essential roles in relaying signals downstream of activated T and B cell surface antigen receptors in the adaptive immune response and auto-antibody production have also been linked to SLE susceptibility.^153,154^ For instance, risky alleles of SNPs rs2230926 (*TNFAIP3* that encodes TNFα induced protein 3),^155^ rs2476601 (*PTPN22* that encodes protein tyrosine phosphatase non-receptor type 22),^156,157^ rs7829816 (*LYN* that encodes lymphocyte-specific protein tyrosine kinase),^158^ rs10513487 (*BANK* that encodes B cell scaffold protein with ankyrin repeats),^153^ rs7812879 (*BLK* that encodes B-cell receptor-associated protein kinase),^159^ rs340630 (*AFF1* that encodes AF4/FMR2 family member 1),^160^ rs4810485 (*CD40*),^161^ rs3433034 (*CSK* that encodes C-terminal Src kinase),^162^ rs17849502 (*NCF2* that encodes neutrophil cytosolic factor 2),^163^ rs1057233 (*PU.1* that encodes purine rich box-1)^164^ have been reported to alter T and/or B cell activation threshold in the adaptive immune response for enhanced chance of developing SLE.

#### Genetic factors associated with debris clearance ability

As the classical pathway activating complement signaling may help remove apoptotic and damaged cells as well as ICs for reduced risk of developing autoimmunity to nuclear components,^165^ low copy numbers of *C4* and *C1q* or deficiency of these genes are typically associated with increased SLE incidence. It has been estimated that the chance of developing SLE among individuals harboring congenital genetic deficiencies of *C4* increased up to 90%.^127^ Several genetic mutations associated with the complement genes have been documented. For example, the *C4A*-null allele was associated with a doubled SLE susceptibility than either *HLA-B8* or *HLA-DR3*,^166^ where *HLA-B8* and *HLA-DR3* alleles were known to predispose the risk of SLE via influencing early stages of adaptive immune activation.^167^ *C1qA* gene deficient mice have a greater chance of accumulating apoptotic bodies in the kidney and developing auto-antibodies to nuclear antigens.^168^ Also, C1q was shown protective against SLE via directing the immune stimulatory complexes to monocytes rather than DCs that secrete the pro-inflammatory cytokine IFNα,^169^ and through modulating CD8^+^ T cell mitochondrial metabolism for reduced immune response to self-antigens.^170^ In addition, multiple high-frequency low-risk genetic polymorphisms residing in 1q36 have been linked to genes encoding components of the complement system and been considered responsible for debris clearance.^171–173^

### Epigenetic factors predisposing SLE

Epigenetic factors predisposing SLE include, primarily, miRNA, DNA methylation, and histone modification.

#### miRNA

miRNAs can act similarly as the TFs or interplay with TFs to cooperatively regulate the expression of target genes.^174^ Numerous studies have demonstrated the biological and clinical relevance of miRNAs in SLE. For instance, 42 differentially expressed miRNAs were identified from the PBMCs of SLE patients, among which 7 miRNAs (i.e., miRNA-10a, miRNA-130b, miRNA-134, miRNA-146a, miRNA-31, miRNA-95, miRNA-99a) were more than 6 folds lower in the diseased group as compared with the control.^175^ 4 miRNAs (i.e., miRNA-371-5p, miRNA-423-5p, miRNA-638, miRNA-663) and 1 miRNA (i.e., miRNA-1224-3p) were found to be up- and down-ward regulated, respectively, in LN cells from a study investigating the miRNA profiles of Epstein-Barr virus (EBV)-infected B cells and frozen PMBCs from LN patients and unafflicted controls of different racial groups (i.e., American of African and European origins).^176^ While being up-regulated in this study, miRNA-423 and miRNA-663 were reported to be down-regulated in another study examining the miRNA profiles of kidney biopsy specimen, where 66 miRNAs were identified differentially expressed in LN cells.^177^ Focusing on T and B lymphocytes, it was reported that 3 miRNAs (i.e., miRNA-21, miRNA-25, miRNA-106b) were up-regulated in both T and B cells of SLE patients, 8 miRNAs (i.e., let-7a, let-7d, let-7g, miRNA-148a, miRNA-148b, miRNA-196a, miRNA-296, miRNA-324-3p) and 4 miRNAs (i.e., miRNA-15a, miRNA-16, miRNA-150, miRNA-155) showed altered expression in solely T and B cells of SLE patients, respectively, from a study analyzing the expression of 365 miRNAs in PBMCs from 34 SLE patients and 20 healthy individuals.^178^ Another group reported 11 miRNAs differentially expressed in CD4^+^ T cells from SLE patients, out of which 6 (i.e., miRNA-1246, miRNA-126, miRNA-1308, miRNA-574-5p, miRNA-638, miRNA-7) were up-regulated and 5 (i.e., miRNA-142-3p, miRNA-142-59, miRNA-155, miRNA-197, miRNA-31) were down-regulated.^179^ Although numerous miRNAs have been found dysregulated in human SLE patients or pre-clinical animal models, quite few miRNAs and their altered profilings were overlapping across studies, some of which even showed inconsistent patterns. This can be, at least, partially explained by the diversified manifestations and activities of SLE, as well as its heterogeneity regarding, e.g., ethical race and medication history. This makes miRNAs showing conserved alteration profiles among SLE patients highly valuable from the perspectives of both diagnosis and therapeutics.

##### miRNA-155: promoting SLE

Multiple lines of evidence have indicated that miRNA-155 is activated in response to the stimulation of TLR ligands,^180,181^ and up-regulated miRNA-155 is associated with activated TLR signaling. Multiple targets of miRNA-155 have been shown with critical roles during TLR signaling. For instance, suppressor of cytokine signaling (SOCS1), being a target of miRNA-155, participated in IFN-mediated antiviral response and thus attenuated viral propagation in macrophages.^182^ Inositol polyphosphate-5-phosphatase D (INPP5D), another target of miRNA-155, negatively regulated TLR4 signaling in response to lipopolysaccharide (LPS) stimulation.^183^ Myeloid differentiation primary response protein 88 (MyD88), being targeted by miRNA-155, acted as a vital adapter molecule in TLR signaling.^184^ TGF-β-activated kinase 1 (TAK1)-binding protein 2 (TAB2) is a direct target of miRNA-155 that activated TLR-mediated nuclear factor kappa B (NF-κB) in LPS-activated DCs^185^ and plasmacytoid DCs.^186^ Besides, miRNA-155 played an essential role in dictating the antigen-presenting activity of DCs by suppressing the expression of PU.1 and cellular oncogene fos (c-Fos), two TFs with critical functionalities in regulating DC maturation,^187^ and defect DCs were associated with attenuated immune response.^188,189^

miRNA-155 also modulates the cytokine microenvironment by altering the distribution profiles between Th1 and Th2 cells. Specifically, altered Th1 function, skewed Th2 differentiation, and defective B-cell class switching were observed in mice carrying miRNA-155 deficiency as a result of abnormal secretion of cytokines such as TNFα, IL4 and IL10, and IFNγ.^190,191^ In addition, miRNA-155 affects cytokine homeostasis by changing the distribution between Th17 and Treg cells. For instance, miRNA-155 was negatively involved in Treg cell-mediated tolerance, as miRNA-155 depletion resulted in enhanced Treg-mediated immune suppression;^192^ and miRNA-155 knockout mice exhibited mass loss of Th17 cells coupled with marked reduction of inflammatory Th17 cytokines.^193^

##### miRNA-146a: suppressing SLE

On the opposite to miRNA-155, miRNA-146a level was negatively associated with the risk of SLE,^194^ substantially down-regulated in SLE patients, and adversely correlated with SLE activity.^195,196^

miRNA-146a functions as a strong negative regulator of TLR signaling by repressing TNF receptor-associated factor 6 (TRAF6) and IRAK1.^197^ miRNA-146a expression was inversely correlated with TNFα production, rendering cells tolerant and cross-tolerant to TLR stimulus.^198,199^ In line with these, miRNA-146a was found capable of regulating the production of type I IFNs (i.e., IFNα, IFN-β).^175^ Specifically, in SLE patients lack of miRNA-146a expression, aberrant accumulation of the targeted proteins of miRNA-146a (such as STAT1, IRF5, TRAF6, and IRAK1) led to altered activation of the IFN pathway;^175^ and exogenously introducing miRNA-146a into PBMCs from SLE patients dramatically alleviated the overtly activated type I IFN signaling.^175^ The latter can be evidenced by the approximately 75% reduction on the transcriptional levels of 3 IFN-inducible genes, i.e., IFN-induced protein with tetratricopeptide repeats 3 (IFIT3), myxovirus resistance 1 (MX1), and 2′,5′-oligoadenylate synthetase 1 (OAS1).^175^

Similar to miRNA-155, miRNA-146a regulates the cytokine microenvironment by affecting both the Th1/Th2 and Th17/Treg balances. Specifically, miRNA-146a was highly expressed in Treg cells in response to T cell receptor (TCR) activation, leading to impaired IFNγ-dependent Th1 activity and IL2 secretion, the process of which involved STAT1.^200,201^

##### Other miRNAs

miRNAs modulating immune response

Numerous evidence has supported the notion that miRNAs are essential players in TLR signaling for stimulating immune response. For example, miRNA-124 suppressed macrophage activation by repressing the expression of CCAAT/enhancer-binding protein alpha (C/EBPα),^202^ miRNA-126 reduced the expression of PU.1 and thus TLR activation in allergic asthma.^203^ It is noteworthy that the same miRNAs may not act uniformly under distinct cellular contexts or pathologic conditions. For instance, let-7i, capable of negatively regulating TLR4 expression, was down-regulated in human cholangiocytes^204^ but up-regulated in DCs in response to LPS stimulation.^205^

miRNAs play critical roles in the regulation of T cell development. For example, miRNA-184 restricted the activation of CD4^+^ T cells during the early adaptive immune response and thus limited the production of IL2 by targeting nuclear factor of activated T cells 1 (NFAT1);^206^ miRNA-181c showed a similar function, the ectopic expression of which suppressed IL2 expression and thus reduced the proliferation of activated CD4^+^ T cells;^207^ furthermore, IL2 induced the expression of miRNA-182, leading to inhibited activity of Forkhead box O1(FOXO1) and T cell clonal expansion.^208^ miRNAs also actively participate in B cell development. For instance, miRNA-150 dramatically impaired B cell expansion via suppressing the critical TF required for B cell differentiation, i.e., c-Myb;^209^ miRNA-181a promoted B cell differentiation in mouse bone marrow when ectopically expressed in B cell progenitors^210^ besides regulating TCR signaling in immature T cells.^211^ It has been documented that transplanting bone marrow cells over-expressing miRNA-181a to lethally irradiated mice promoted the growth of CD19^+^ B cells and reduced the amount of CD8^+^ T cells.^210^

miRNAs modulating cytokine microenvironment

Besides targeting TLR signaling pathways and regulating immune cell development, multiple lines of evidence have unambiguously supported the essential roles of miRNAs played in modulating cytokine homeostasis. It has been well documented that altered secretion profiles of cytokines such as IL2, IL6, IL10, and regulated on activation normal T cell expressed and secreted (RANTES) play crucial roles in SLE development, and miRNAs participate in SLE development through modulating the production of these primary cytokines. For example, the level of RANTES was documented to be abnormally over-represented in the blood sera of SLE patients, whereas that of IL2 was reported to be significantly lower in lupus T cells. Under-expressed miRNA-31 contributed to the decreased IL2 expression in PBMCs or lupus T cells.^175,179^ miRNA-142-3p was highly induced in DCs in response to LPS stimulation, leading to suppressed IL6 production.^212^ Up-regulated miRNA-21 expression has been positively associated with SLE activity, reduced level of which in SLE CD4^+^ T cells led to decreased IL10 production.^178^ Through characterizing miRNAs lowly expressed among SLE patients, miRNA-125a was found capable of reducing T cell-mediated RANTES production via targeting its TF, i.e., Kruppel-like factor 13 (KLF13), and exogenously introducing miRNA-125a into the T cells of SLE patients resulted in significantly alleviated up-regulation on RANTES expression and SLE severity.^213^

#### DNA methylation

DNA methylation level has been considered to be lower in SLE patients or lupus animal models.^214,215^ Specifically, DNA extracted from the CD4^+^ T cells of SLE patients was hypomethylated,^215^ and adoptive transfer of T cells pre-treated with DNA methylation inhibitors induced SLE symptoms in unirradiated syngeneic mice.^216,217^ A clinical study involving 1521 Chinese and European SLE patients, along with healthy controls and patients with other autoimmune diseases such as rheumatoid arthritis (RA) and primary Sjögren’s syndrome (pSS), revealed that SLE patients are characteristic of hypomethylation at two CpG sites within the promoter region of *IFI44L*, SLE patients with renal involvement displayed even lower methylation levels at these sites, and the methylation levels increased among SLE carriers during remission.^218^ These suggested that the methylation level of the promoter region of *IFI44L* may serve as the blood biomarker for SLE prognosis and diagnosis.^218^ In Feb 2024, the world’s first innovative *IFI44L* gene methylation detection product was approved by National Medical Products Administration (NMPA) of China for SLE prognosis prior to the onset of vital organ damage.^219^ This may be attributable to the inhibited inheritance of DNA methylation profiles during mitosis in response to perturbations such as aging and diet^220–222^ that involves the participation of multiple miRNAs such as miRNA-126, miRNA-148a and miRNA-21.^179,223^

DNA methylation patterns are regulated by methyltransferases, including DNA (cytosine-5)-methyltransferase 1 (DNMT1), DNMT3A, DNMT3B, and DNMT3L.^224,225^ While, DNMT1 maintains DNA methylation profiles, DNMT3A and DNMT3B introduce de novo DNA methylation, and DNMT3L assists the functionalities of DNMT3A and DNMT3B.^226,227^ It has been reported that miRNAs such as miRNA-126^179^ and miRNA-148a^223^ regulated the levels of DMNT1 in the T cells of SLE patients. Specifically, miRNA-148a and miRNA-21 were robustly up-regulated in CD4^+^ T cells from SLE patients lupus-prone MRL/lpr mice, giving rise to DNA hypomethylation via suppressing DNMT1 expression;^223^ and miRNA-126 inhibited DNA methylation in CD4^+^ T cells of SLE patients by binding to the 3’ untranslated region (3’ UTR) of DNMT1.^179^ In addition, defective ERK pathway in T cells negatively affected DNMT1 expression and enhanced the development of anti-dsDNA antibodies in transgenic mice,^228^ suggesting the involvement of suppressed ERK signaling in priming DNA hypomethylation among SLE carriers.

#### Histone modification

Histone 3 (H3) and Histone 4 (H4) hypoacetylation and site-specific histone methylation alterations were found in the CD4^+^ T cells from SLE patients and MRL-lpr/lpr mice splenocytes.^229,230^ It has been reported that the histone deacetylase inhibitor trichostatin A (TSA) can restore skewed expression of IL10, IFNγ and CD154 in lupus T cells,^231^ and treating MRL-lpr/lpr mice with histone deacetylase inhibitors TSA and suberoylanilide hydroxamic acid (SAHA) reduced the section of IL6, IL10, IL12, and IFNγ.^232,233^ These findings implicated that histone modification variation contribute to the modulation of cytokine distribution in SLE pathogenesis.

### Hormonal factors predisposing SLE

Female hormones such as estrogen and prolactin contribute to the activation of the immune system and thus predispose the prevalence of SLE, leading to the extreme female predominance among SLE carriers. Specifically, estrogen functions by skewing the cytokine microenvironment to favor Th2, prolactin acts via activating the immune response. Progesterone, on the other hand, represents a protective factor of SLE by damping the immune activating signals.

#### Estrogen

Estrogens have been reported to potentiate Th2-mediated diseases including SLE by inhibiting the production of Th1 pro-inflammatory cytokines such as IL12, TNFα and IFN${\rm{\gamma }}$, and stimulating the secretion of Th2 anti-inflammatory cytokines such as IL4, IL10, and TGFβ.^68^

Estrogens include estrone (E1), estradiol (E2), and estriol (E3), with E2 being the primary biologically active estrogen. Estrogen receptors (ERs), both nuclear and membrane bound, have been identified in various types of cells involved in the innate and adaptive immune responses.^234^ While the nuclear ERs include ERα and ERβ, the membrane-bound ER is the G-protein-coupled estrogen receptor (GPER).^235^ ERs contain three functional domains, i.e., trans-activation domain, DNA-binding domain, and ligand-binding domain.^236^ During nuclear ER signaling, ERα and ERβ typically act in an opposite fashion in response to E2 treatment and regulate almost distinct sets of genes, with only 38 out of 228 genes being regulated by both ERα and ERβ.^237^ Nuclear ER signaling can be long-term and manifest genomic information via transcriptionally regulating a plethora of factors including, e.g., cytokines such as IFNs and signaling pathways such as JAK/STAT signaling.^234,238,239^ Different from nuclear ER signaling, GPER-mediated signaling is rapid and nongenomic. Specifically, the signal transduction cascade is initiated via intracellular calcium and cyclic adenosine monophosphate (cAMP) induction, and leads to activated phosphoinositide 3 kinase (PI3K)/ AKT and mitogen activated protein kinase (MAPK)/ extracellular signal regulated kinase (ERK) signaling.^235^

Estrogen contributes to the polarization of the cytokine environment to the Th2 state, where a shift of the balance between Th1 and Th2 subsets to Th2 dominance is characteristic of SLE. Specifically, low doses of estrogen promote Th1 responses for increased cellular immunity, and high doses of estrogen elevate Th2 responses for stronger humoral immune responses.^240,241^ This effect of estrogens is achieved via altering the Th cytokine profile from a Th1-dominant state (IL12, IFNγ, TNFα) to a Th2-dominant profile (IL4, IL6, IL10, TGFβ).^68^ For instance, E2 as well as E1 and E3 have been shown to stimulate TNFα secretion at low concentrations and inhibit it at high concentrations,^242^ the effect of which on IL10 production was shown to be the opposite.^243^

#### Prolactin

Prolactin, with increased levels detected in the serum of SLE patients, functions as both a hormone and a cytokine. Prolactin has been shown capable of stimulating almost all primary players in the innate and adaptive immune responses such as T cells, B cells, DCs, natural killer (NK) cells, macrophages, neutrophils, and hematopoietic stem cells according to a collection of in vitro, in vivo and clinical evidences. Though prolactin disruption is not essential for the normal development and functionality of the immune system,^244^ prolactin can synergize with IL2 in B cell activation and differentiation.^245^ In addition, the prolactin receptor is expressed on human immune cells including T and B lymphocytes and monocytes,^246^ suggestive of its promotive roles in SLE development.

#### Progesterone

Pregnancy-associated changes in progesterone signaling has been considered important for innate immune surveillance and tolerogenic response, rendering progesteron a protective factor of SLE. Specifically, progesterone reduces the secretion of proinflammatory cytokines such as TNFα, IL1β and IL12, leading to attenuated activities of primary players involved in both the innate and adaptive immune responses such as macrophages, DCs, CD4^+^ and CD8^+^ T cells.^239^

### Extrinsic factors predisposing SLE

The contribution of extrinsic risk factors to SLE susceptibility increases with age, as a greater contribution of known SLE genetic risk alleles (especially residing in non-*HLA* genes) were found among children who developed SLE than adult SLE patients.^247,248^

Extrinsic trigger of SLE can be primarily classified into three categories based on their effects on SLE pathogenesis, i.e., events introducing immune activators, perturbing cytokine microenvironment homeostasis, and inducing inflammation.^8^

#### Events introducing immunoreactants

EBV infection can increase the amount of EBV nucleic acids in the blood of SLE patients,^249^ which activates the innate immunity and B cell differentiation by expressing type I IFNs and stimulating the production of autoantibodies specific for EBV-encoded proteins.^250,251^ It is worth noting that EBV infection predisposes to SLE development but not vice versa, as the serum anti-EBV capsid antigen IgG levels of SLE patients were significantly higher than healthy individuals that did not apply to anti-EBV nuclear antigen.^252^ The mRNA/DNA vaccines may also induce SLE. It has been reported that the application of mRNA or DNA vaccines against the coronavirus disease (COVID-19) has been shown capable of causing new or relapsed onset of SLE.^253–256^ A boost in spike protein-specific CD4^+^ Th1 and CD8^+^ T cell responses were detected after the use of AZD1222 (i.e., a DNA COVID-19 vaccine),^257^ the mechanism of which could be attributed to activated TLRs followed by induced type I IFNs-mediated signaling. Additionally, agonists of TLR7 and/or TLR9 have been oftenly supplemented as the adjuvants in mRNA/DNA COVID-19 vaccines for enhanced immunity,^258,259^ further aggregating the development of SLE.

UV light irradiation may activate the autoimmune response via generating nucleic acid fragments, attributing to its breakage role on DNA strands.^46^ A clinical study examined the sensitivities of 100 SLE patients to UV radiation, where 93% patients showed abnormal reaction to UV and visible light including, e.g., superficial perivascular lymphocytic infiltrate and deposition of immunoreactants such as IgG and C3.^260^

Medications with pro-inflammatory roles may also induce SLE that can be manifested as vasodilation and hypotension. For instance, hydralazine (a vasodilator) and procainamide (an anti-arrhythmic agent) can trigger SLE via forming neutrophil extracellular trap (NET)^261,262^ that can function as the auto-antigens due to DNA, histones and neutrophil proteins it contains.^263^ Excessive secretion of pro-inflammatory cytokines such as IFNα (used for treating Hepatitis B/C) aggravates SLE.^264–266^

Habits such as tobacco smoking is a known risk factor for SLE in a dose-dependent manner as it is a stimulus capable of inducing nonspecific inflammation and, thus, autoimmune responses among SLE carriers.^267^

#### Events skewing cytokine microenvironment

Some medications may induce SLE, though the symptoms may be milder than idiopathic SLE. The mechanisms-of-action may be attributed to their roles in skewing the cytokine microenvironment. For instance, carbamazepine, an anticonvulsive agent traditionally used for treating epileps and neuropathic pain, can increase IL5 secretion that marks Th2 production, attributing to the terminal metabolite acridine it produced.^268^ Sulfasalazine, used for treating rheumatoid arthritis, can skew the cytokine microenvironment to Th2-dominant state by suppressing IL12 production in macrophages.^269^ Another example refers to antibodies against TNFα that have been used as immunosuppressors in the treatment of autoimmune or inflammatory diseases. Infliximab, an anti-TNFα antibody, induced the production of anti-dsDNA antibody and SLE among more rheumatoid arthritis patients.^270,271^ Similarly, hydralazine, procainamide and other DNA methyltransferase inhibitors such as 5-azacytidine may turn CD4^+^ T cells autoreactive to spontaneously lysed syngeneic macrophages and produce more IL4/6 and IFNγ to induce SLE.^216,220^ The combinatorial use of minocycline (a semi-synthetic tetracycline-class broad-spectrum antiboitic) with bone marrow derived mesenchymal stem cells (MSCs) in treating autoimmune encephalomyelitis, though having achieved desirable therapeutic effects in an autoimmune encephalomyelitic mice model, may increase the risk of developing SLE syndromes as a result of suppressed production of IFNγ and TNFα as well as increased generation of IL4 and IL10.^272^ Paradoxically, minocycline decreased C-C motif chemokine ligand 22 (CCL22) production from macrophage type 2 for reduced Th2 recruitment to the lesion,^273^ implicating the importance of cytokine microenvironment homeostasis in preventing autoimmune syndromes that is dictated, at least partially, by the type and cytokine profile of the syndrome as well as the medication strategy being applied.

## Preventive strategies for SLE management

SLE could be initiated and accelerated by complicated dynamic interplays between intrinsic and extrinsic factors. Specifically, once individuals possessing SLE genetic, epigenetic or hormonal risk factors are chronically exposed to extrinsic risk factors, accelerated disease onset and deterioration may occur. As intrinsic risk factors including those at the genetic, epigenetic, hormonal levels are difficult to control, we focus on preventive approaches against the extrinsic risk factors in this section.

Extrinsic factors can be environmental situations such as virus infection, UV light irradiation, heavy metal exposure, air pollution and silica, habitual factors such as unhealthy diet, cigarette smoking, lack of physical exercises and sleep deprivation, physiological conditions such as comorbidities, obesity and pregnancy, and psychological factors such as trauma and stress.^274–276^ Current conceptions on a healthy lifestyle with a lower risk of developing SLE overall include, e.g., a healthy eating habit (i.e., top 40% of the Alternative Healthy Eating Index), no smoking, moderate alcohol consumption (i.e., no less than 5 gm per day), regular exercise (performing at least 19 metabolic equivalent hours of exercise per week), and fitness (i.e., body mass index below 25 kg/m^2^).^277^ Each of these preventive recommendations has a 19% additive value in reducing the chance of developing SLE especially among anti-dsDNA antibody positive patients; and the cumulative risk of having SLE can be reduced to half of those with the poorest behavior for individuals keeping the best adherence to the healthy lifestyle. This implicates that extrinsic factors may act synergistically to influence the risk of SLE, and SLE may be prevented by, e.g., altering the lifestyle among other extrinsic factors.

Preventive strategies can be classified into three stages, i.e., primary prevention, secondary prevention, tertiary prevention, which should be adopted as early as possible to prevent the development, exacerbation and progression of SLE, respectively. This especially holds true for individuals who have already been prognosed at a high risk of developing SLE or diagnosed with SLE.

**Fig. 5:**
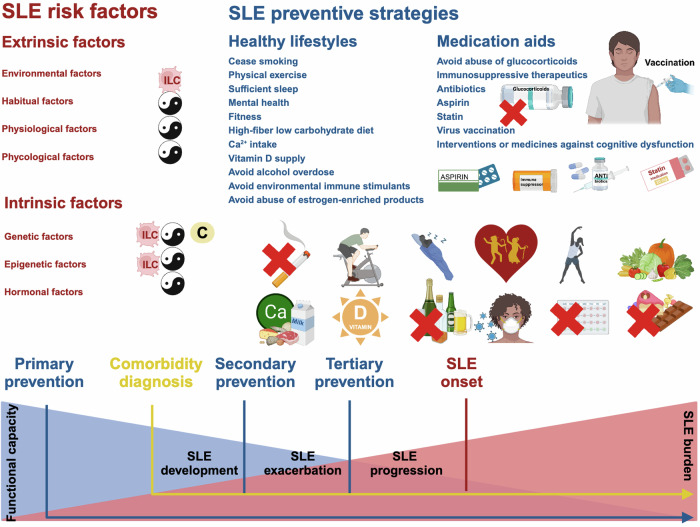
Risk factors and preventive strategies against SLE development, exacerbation, progression. Risk factors predisposing SLE can be classified into intrinsic and extrinsic factors (in red). Intrinsic factors can occur at genetic, epigenetic and hormonal levels. Extrinsic factors can be environmental, habitual, physiological and phycological. Among these extrinsic factors, SLE comorbidities from the category of physiological factors include cancers, cardiovascular diseases, bone diseases and neuropsychiatric diseases. As intrinsic SLE predisposing factors are difficult to control, preventive strategies against each extrinsic risk factors are listed (in blue). By summarizing preventive strategies against SLE and its comorbidities, 11 recommendations on the lifestyles (i.e., cease smoking, physical exercise, sufficient sleep, mental health, fitness, High-fiber low carbohydrate diet, Ca^2+^ intake, vitamin D supply, avoid alcohol overdose, avoid environmental immune stimulants, avoid abuse of estrogen-enriched products) and 7 advises on medication aids (avoid abuse of glucocorticoids, immunosuppressive therapeutics, antibiotics, aspirin, statin, virus vaccination, interventions of medicines against cognitive dysfunction) are given. SLE preventive strategies can be classified into three stages, i.e., primary prevention against SLE development, secondary prevention against SLE exacerbation, tertiary prevention against SLE progression. The prevention of SLE comorbidities should start at the time of diagnosis. Applying preventive strategies as early as possible during the disease course can help to avoid organ damage, which is a major trigger of systematic functional decline. Primary prevention recommendations are emphasized using cartoons

Following the rationals of mechanisms-of-action, preventive strategies can be classified into approaches against events introducing immune stimulants, and skewing the cytokine microenvironment (Fig. 5).

### Prevention of SLE

Preventive strategies recommended below are categorized by the pathogenic stages leading to SLE. These approaches are suitable to individuals diagnosed with high risks of developing SLE for prevented disease onset, and to SLE patients for alleviated disease symptom or delayed disease progression.

It is worth to highlight that intrinsic and extrinsic factors especially biomarkers at the genetic and epigenetic levels as aforementioned can aid in SLE prevention and early intervention, since they could help identify individuals with the high risk of developing SLE that require early adoption of prevention strategies.

Also deserves emphasizing is that there is current no consensus on strategies for SLE prevention given the complexity and heterogeneity of the etiology of this disease. Besides, the full spectrum of preventive strategies against SLE is not completely uncovered, personalized prognosis and prevention modality design are not yet available. Thus, approaches beneficial to one individual may not be optimal or even effective to another.

#### Prevention against events introducing immune stimulants

As an important type of extrinsic factors, environmental situations with SLE-predisposing roles such as EBV infection, exposure to UV light, heavy metals such as mercury,^278^ agricultural pesticides,^278–280^ air pollution, crystalline silica dust,^281–284^ and other respiratory particulates^285,286^ largely act via exposing individuals with immune stimulants. These factors function primarily by stimulating cellular necrosis and secreting intracellular antigens for up-regulated IFN levels and promoted inflammation. Take EBV infection as an example, it functions by releasing EBV-encoded small RNA from infected cells that induces type I IFN signaling (in particular TLR3-mediated signals),^287^ with a substantially higher seroprevalence of anti-viral capsid antigens IgG and antibodies against early antigens being observed among SLE patients as compared with non-diseased controls according to a meta-analysis involving 25 case-control studies.^252^ Additionally, UVB radiation, another important trigger of SLE, led to a significant rise in type I IFN signaling and prolonged activation of T cells both in lupus-prone mice and among SLE patients.^288–290^ A randomized, vehicle-controlled, double-blind clinical study including 25 CLE patients revealed the photoprotective effects of broad-spectrum sunscreen.^291^ There also exists clinical evidence supporting the benefits of using sunscreen for preventing the onset or worsening of SLE.^292^ Yet it is worth noting that the association of UV radiation with SLE risk is convolved with its positive roles in vitamin D3 synthesis^293^ that may, to some extent, reduces SLE risk.^294^ Therefore, keeping away from immune stimulants such as virus infection, overt UV light exposure and any environmental pollutant is highly advised among individuals genetically predisposed with SLE.

Receiving vaccination to elevate the thresholds of responding to environmental stimuli may represent another useful strategy. Yet, the efficacy of vaccination in reducing SLE risk remains to be elucidated.^295^ This is because that vaccines may contain elements such as molecular mimicry, auto-antibodies and adjuvants that can potentially trigger autoimmune responses towards accelerated SLE. Evidences supporting this rational include a series of reports on the new-onset of autoimmune diseases including autoimmune hepatitis disease,^296^ autoimmune thrombotic events,^297^ rheumatoid arthritis,^298^ immunoglobulin A vasculitis,^299^ Guillain-Barré Syndrome and,^300^ importantly, SLE,^255,301^ after getting vaccinated against COVID-19. Also, a clinical case on the transition of cutaneous lupus erythematosus (CLE) to SLE after COVID-19 vaccination has been identified.^302^ However, these reports are largely from case reports or cross-sectional studies representing temporal associations and are not from vaccinations against EBV. On the other hand, therapeutic EBV vaccines have been considered promising in treating cancers with acceptable toxicity.^303^ Take together, establishing vaccines against EBV or any other immune stimulants for SLE prevention may be worthwhile to try but requires intensive investigations and clinical monitoring on the possible adverse effects accompanied besides the efficacy.

#### Prevention against events skewing cytokine microenvironment

Many lifestyles and physiological conditions associated with SLE predisposition can increase levels of pro-inflammatory cytokines, leading to skewed cytokine microenvironment.

Cigarette smoking has been associated with increased risk of developing anti-dsDNA antibody positive SLE than non-smokers by several clinical investigations,^267,304,305^ linked to augmented autoreactive B cells by a clinical evidence based meta-analysis,^267^ and associated with induced pulmonary ANA in the lungs of exposed mice.^306^ In addition, a cross-sectional study containing 105 smokers revealed the positive association of cigarette smoking with cumulative chronic damage in SLE patients and its deleterious effects on lupus morbidity.^307^ This may be attributed to the toxic components of the cigarette that can damage DNA to form immunogenic DNA adducts for promoted production of anti-dsDNA antibody and pro-inflammatory cytokines.^308,309^ In particular, smoking can increase the expression of B lymphocyte stimulator (BLyS) that is a soluble ligand of the TNF cytokine family,^306^ TNFα and IL6.^310^ Among positive ANA women, elevated BLyS and lower IL10 (an anti-inflammatory cytokine) levels were identified among frequent smokers.^311^ Therefore, cease smoking is highly recommend especially for those with genetic preposition to SLE.

Sleep-deprivation (less than 7 h per night) has also been associated with an earlier SLE onset and accelerated auto-antibody production from both pre-clinical animal models and clinical observations.^312–314^ In particular, from a prospective study involving 436 participants having relatives already diagnosed with SLE, the chance of developing SLE among individuals sleeping less than seven hours per day was 2.8-folds of that among the rest with statistical significance.^314^ In a larger trial involving 186072 women, chronic low sleep duration (i.e., less than 5 h/day) was associated with increased SLE risk (adjusted HR = 2.47) with confounding factors shiftwork, bodily pain and depression being adjusted.^315^ At the molecular level, insufficient sleep has been shown to be capable of increasing levels of pro-inflammatory IL6 and TNFα, leading to impaired functionalities of Treg cells, imbalanced cytokine milieu, systematic inflammation and ultimately endangered self-immune tolerance.^278^ Thus, keeping sufficient sleep (e.g., seven to eight hours per day) and avoiding night or rotating shifts are recommended for individuals of all ages predisposed with SLE.

Adipose tissue especially visceral fat can secrete pro-inflammatory cytokines such as IL6 and possess high levels of TNFα receptor 2 (TNFR2) as compared with non-obese individuals.^316^ In addition, both TNFα and IL6 have been implicated with critical roles in the modulation of insulin resistance.^317^ In a Mendelian randomization study, 3 out of 25 identified cytokines associated with obesity (i.e., CTACK, IL18 and SCGFb) were related to SLE, and 2 out of 18 characterized cytokines associated with SLE (i.e., IP10 and MIP1B) were also linked to obesity,^318^ implicating the intermediary role of these cytokines in the association between obesity and SLE. Thus, keeping fitness may be an effective preventive approach against SLE. To avoid obesity, one might be recommended to take food enriched with fibers and/or adhere to a low-carbohydrate or ketogenic diet that have been shown associated with reduced risk of developing SLE via decreasing glucose-induced inflammation and preventing obesity.^319,320^

Women who had experienced physical and emotional abuse during childhood exhibit higher chances of developing SLE as compared with those who had not.^321,322^ In an analysis of data from 36152 black women, both physical and sexual abuse during childhood were statistically associated with increased SLE incidence, where other factors including alcohol consumption, smoking, body mass index, oral contraceptive use, age at menarche, and parental education were adjusted. The HR of women with more than 2 episodes of sexual abuse was 2.51-folds of the control group, implicating the significant promoting role of physical and emotional abuse during childhood on SLE risk.^321^ In consistent with this, psychosocial trauma and associated post-traumatic stress disorder may lead to autoimmune diseases among women including SLE.^323^ In a cross-sectional analysis of data from the California Lupus Epidemiology Study involving 242 SLE adult patients, more recent stressful events were statistically associated with higher stress among people having trauma and adverse childhood experiences, and positive psychosocial factors showed an opposite association.^324^ These results suggested the mitigating role of a positive mental state in perceived stress that may improve outcomes in SLE, even among individuals suffered from prior trauma and thus more vulnerable to recent stressful events. Besides, depression has been recognized as an important risk factor of SLE among females.^325^ In a cross-sectional study conducted among 85 Chinese SLE patients, a robust correlation was identified between SLE disease activity and depression that was weakened during disease remission; besides, the presence of mucosal ulcer significantly increased the risk of developing depression among these patients.^326^ These results supported the negative role of depression on SLE risk. Mechanically, Th2 cytokines are generated to protect the organism from over-production of the pro-inflammatory Th1 cytokines in response to stress; under certain circumstances, stress hormones may promote inflammation via inducing the production of cytokines such as IL6 and TNFα as well as through activating pathways involving corticotropin-releasing hormone and substance P induced histamine.^327^ The stress system, being either hyperactive or hypoactive, may contribute to SLE pathogenesis, the process of which involves an abnormal interface between the neuroendocrine and the immune systems.^327^ Thus, conditions associated with dramatic changes in the stress system such as acute or chronic stress, pregnancy and the postpartum period may potentiate SLE via altering the Th1/Th2 cytokine homeostasis.^327^ These have collectively explained the predisposing roles of emotional depression and stress on SLE onset, and suggested the importance of keeping the mental health among individuals at high risks of developing SLE.

Estrogen has been considered capable of up-regulating the expression of several genes involved in B cell activation and survival such as those encoding CD22, Src homology 2 (SH2) domain-containing phosphatase 1 (SHP1), B-cell lymphoma 2 (BCL2), and vascular cell adhesion molecule (VCAM),^328^ explaining the increased SLE susceptibilities of oral contraceptive pill (with ethinyl estradiol being the major ingredient) users and individuals receiving hormone replacement therapy.^329–331^ In a two-sample Mendelian randomization analysis of a GWAS data, a negative causal relationship was identified between age at menarche and SLE, suggesting the positive role of estrogen played in SLE risk predisposition.^332^ Thus, reducing the use of estrogen-enriched products is highly recommended for women with genetic SLE predisposition.

### Prevention of SLE comorbidities

SLE comorbidities should be prevented or treated, which is also of the paramount importance as these conditions can lead to the ultimate death in a considerable proportion of SLE patients.^333^ Diseases typically accompanied with SLE include neoplasms especially hematological cancers, cardiovascular diseases, bone diseases such as osteonecrosis, and neuropsychiatric manifestations such as cognitive dysfunction.

#### Cancers

SLE-associated cancers can mostly be induced by virus infection. The International Agency for Research on Cancer (IARC) has implicated virus infection as one primary etiological factor leading to cancers that contributes to around 15% of new cancer annual incidence. Viruses capable of inducing cancers are defined as Group I ‘carcinogenic to humans’ by IARC, which include, e.g., human papillomavirus (HPV), hepatitis B virus (HBV), hepatitis C virus (HCV), EBV, Kaposi’s sarcoma herpesvirus (KSHV), Merkel cell polyomavirus (MCPyV), human immunodeficiency virus (HIV), human T-cell lymphotropic virus 1 (HTLV1), simian virus 40 (SV40).^334–337^ In particular, approximately 10% diagnosed cancer cases are induced by virus infection, among which 4.5% are caused by HPV infections.^338^

Among HPV-associated transformed conditions, high risk was identified for epithelial carcinoma in situ of the uterine cervix, vaginal cancer, anal cancer, non-melanoma skin cancer, bladder cancer, liver cancer, lung cancer, and lymphoma especially Hodgkin’s lymphoma.^339–343^ The association between HPV infection and cancer incidence has been proposed to be attributable to the chronic immune activation triggered by persistent virus infection and/or the inefficiency of the immune system to clear off HPV infection.^341^ Therefore, frequent HPV screening, vaccination following gender- and age- associated recommendations, therapeutics removing immune stimulants leading to over-activated immune responses, and medications attenuating chronic over-activated immune system are all helpful for preventing the occurrence and deterioration of SLE-associated cancers.

Specifically, regular gynecological screening, such as the Papanicolaou (PAP) test for women aged 21-30 years old every 3 years and the PAP test combined with the HPV test for women aged 30-65 years old every 5 years, are heavily recommended. Along with the examination, HPV vaccination is suggested among females aged between 21 and 65.^339^

The traditional strategy used to remove immune stimulants such as bacterial infections is to apply antibiotics. For instance, trimethoprim-sulfamethoxazole(TMP-SMX) is a combinatorial antibiotic used for infection treatment, which has been used as a prophylactic agent for patients showing a low T cell activity (i.e., CD4^+^ cells below 200 cells/mm^3^).^344^ However, co-administration of TMP and SMX is not recommended due to its notable adverse effects especially for the increased risk for hematologic toxicity.^345^ In addition, treating patients carrying chronic neutropenia (below 500 cells/mm^3^) with quinolone antibiotic such as levofloxacin (500 mg daily) or ciprofloxacin (500 mg, twice daily), possibly coupled with anti-fungal therapy as well depending on the disease situation, has been recommended as another prophylactic therapy.^346,347^

Medications used for attenuating chronic over-stimulated immune response is to apply immunosuppressive therapies. Agents fell into this category can be sub-classified into three groups based on their effectiveness and toxicity. That is, agents taking actions rapidly but having severe long-term side effects such as glucocorticoids, agents functioning slowly but showing high safety such as hydroxychloroquine, and agents having high non-response rates and unavoidable adverse effects such as cyclosporine A, cyclophosphamide, mycophenolate mofetil, azathioprine, methotrexate, tacrolimus, belimumab and rituximab.^348^ Specifically, glucocorticoids, steroid hormones produced from the cortex of adrenal glands, dampen the innate immune response via attenuating signaling mediated by pattern recognition receptors (PPRs) and resolving the inflammatory response, as well as regulate adaptive immunity through inhibiting T cell activation and B cell production;^349^ hydroxychloroquine attenuates T cell activation via suppressing TLR signaling, inhibiting cytokine production, and reducing the expression of CD154 (a marker of antigen-specific activation of CD4^+^ T cells^350^);^351^ cyclosporine A, a cyclic undecapeptide commonly used as a member of third group, inhibits T cell activation via blocking the synthesis of ILs including IL2.^352^

#### Cardiovascular diseases

Cardiovascular disorders represent another important portfolio of SLE comorbidities. It has been estimated that SLE patients have a 27% higher risk of developing cardiovascular diseases as compared with gender- and age- matched diabetes patients and over double folds the risk of the general population.^353^ Among the diversified types of cardiovascular events, myocardial infarction and stroke are the most commonly observed among SLE carriers, with the hazard ratio of myocardial infarction among SLE patients ranging from 2.6 to 5.1 and that of stroke SLE carriers being 2.1 to 3.3.^354^ In addition to the aforementioned two primary cardiovascular disorders, the chance of developing sub-clinical atherosclerosis also increases among SLE patients, with the atherosclerotic plaques being detected in 25–56% young SLE carriers as compared with 17–70% of individuals from the general cohort with a similar age range.^355–357^

Several SLE-associated with cardiovascular comorbidities have been identified. These include, e.g., SLE-specific genetic risk factors such as the IL19 risk allele associated with a 2.3-fold increased risk of developing myocardial infarction and stroke among SLE carriers due to enhanced levels of IL10 and aPL antibodies,^358^ physiological risk factors such as male gender,^359^ advanced age,^359–361^ and postmenopausal status,^360,361^ lifestyle risk factors such as tobacco smoking,^362^ and medical risk factors such as hypertension,^360^ hypercholesterolemia, diabetes mellitus, and the associated medical interventions.

As genetic and physiological risk factors are difficult to control, preventive strategies against SLE-associated cardiovascular comorbidities largely focus on removing risk factors from the perspectives of the lifestyle and medication. Current recommendations for managing cardiovascular diseases associated with SLE are, in general, similar to the general strategies used for cardiovascular disorder management. These include, e.g., ceasing smoking and taking physical exercises for a healthy lifestyle, and relying on therapeutics for appropriate control of hypertension, hypercholesterolemia, and diabetes.^363,364^ Since it is easy to understand the benefits of forming healthy habits, we focus on the medicinal use for reducing the risk of developing SLE-associated cardiovascular disorders and/or halting their deterioration.

Several medications have been identified to be feasible for preventing cardiovascular diseases associated with SLE. For instance, an appropriate use of statin has been proposed capable of helping prevent cardiovascular diseases among SLE patients given its roles in reducing the low-density lipoprotein levels of the patients.^363^ There also exists evidence supporting a dose-dependent use of aspirin among SLE carriers especially those having at least one risk factor predisposing the onset of cardiovascular diseases due to its anti-thrombotic effects.^365^ Specifically, the anti-thrombotic effects of aspirin are related to its roles in acetylating cyclooxygenase (COX) in the platelets,^366^ and a low-dose aspirin regimen (i.e., over 30 mg/day) can effectively prevent platelet aggregation without affecting the functionalities of endothelial cells.^367^ Yet, a higher dosage and more frequent use of aspirin is required to suppress inflammation, the primary drawback of which is associated with increased risk of developing gastrointestinal syndromes, renal toxicity and hypertension. Taken together, aspirin is recommended as a secondary prevention approach against cardiovascular syndromes associated with SLE but remains controversial to be used for primary cardiovascular disease prevention due to its adverse effects.^368^

It is important to note that several drugs commonly used for treating SLE or certain comorbidities may induce or deteriorate cardiovascular syndromes. For instance, glucocorticoids, capable of dampening both the innate and adaptive immune responses, are known to increase the glucose and cholesterol levels of the blood as well as the blood pressure, which are all risk factors predisposing the onset of cardiovascular disorders.^369,370^ Specifically, several cohort investigations have demonstrated that a long duration use of corticosteroid^371,372^ and a high accumulated corticosteroid dose,^372–379^ are associated with a high incidence of cardiovascular events among SLE carriers.^362,380–382^

#### Bone diseases

Bone diseases especially osteoporosis and fractures frequently occur among SLE carriers, with the frequency ranging from 3 to 40%.^120,383–385^ In particular, independent cohort studies conducted in Taiwan, Sourth Korea, and USA all reported higher incidences of osteoporotic fractures among SLE patients as compared those non-SLE carriers, with the incidence ratio being 1.63 versus 0.92,^386^ 19 versus 6.5,^387^ and 4.32 versus 2.4,^388^ respectively, per 1000 persons per year.

Several risk factors have been alluded to increase the chance of developing osteonecrosis among SLE patients, with the most prominent one being the medicinal use of corticosteroids. It has been recognized that osteonecrosis primarily occurs among SLE patients who have received corticosteroid treatment but not those who have not.^389^ Besides, the prevalence of osteonecrosis among SLE is much higher than in other diseases requiring the use of corticosteroids.^390^ Lots of clinical evidence have implicated that the initial dose, cumulative dose, tapering speed, and treatment duration of corticosteroids, as well as medications used together with corticosteroids all convey significant impact to the incidence, number, volume and location of osteonecrosis.^390^ Thus, avoiding the use or overt dosage of corticosteroid represents an effective strategy for keeping bone health.

Habits such as alcohol abuse and smoking all impose significant negative impacts on the onset of SLE-associated osteonecrosis.^391–393^ Alcohol intake is associated with induced chronic inflammation, impaired bone cell differentiation, and increased adipogenesis, which collectively lead to the development of necrosis including osteonecrosis.^394^ Smoking is capable of inducing osteoblast apoptosis that leads to decreased bone mass.^395^ As preventive strategies, SLE patients are suggested to reduce the amount of alcohol intake and cease smoking, increase calcium (Ca^2+^) and vitamin D intake, and increase physical activities to prevent the onset and deterioration of SLE-associated osteonecrosis. For patients at the risk of fractures, medications against bone resorption or destruction may be considered such as bisphosphonates and denosumab.^396^

Lastly, accumulated evidence have implicated that SLE activity is of the paramount importance in predisposing osteonecrosis.^397–401^ Several clinical studies have supported the positive correlation between the SLEDAI score and the chance of developing osteonecrosis.^398,402^ Several genetic polymorphisms affecting SLE activities have been identified to be positively associated with the risk of developing osteonecrosis. For example, three SNPs from the Korean population (i.e., rs3813946, rs311306, rs17615)^403^ and one SNP from the Chinese population (i.e., rs45573035)^404^ residing in the gene encoding the complement receptor type 2 (CR2) have been reported to be associated with the susceptibility of osteonecrosis. CR2 is a membrane glycoprotein that binds to the degradation debris of C3 and plays critical roles in relaying immune signals such as activating B cells via cooperating with the B cell receptors.^403,405^ Genetic defects in CR2 may lead to impaired debris clearance ability and abnormal B cell signaling, which are both promotive on SLE activity. Another example is that the T/T genotypes from the C1236T and C3435T polymorphisms of the gene encoding the adenosine triphosphate (ATP)-binding cassette subfamily B member 1 (ABCB1) show protective roles against osteonecrosis as compared with the wildtype (C/C) genotypes, where ABCB1 functions by pumping foreign substances out of cells and is lowly expressed among SLE patients.^406–408^ In summary, reduced level of ABCB1 is associated with cells’ decreased ability of removing immune stimulants and consequently increased SLE activity and elevated risk of developing osteonecrosis. Thus, adopting preventive strategies against SLE is highly recommended here for maintaining the bone health.

#### Neuropsychiatric diseases

Neuropsychiatric disorders, ranging from overt neurological syndromes such as psychosis and seizures to subclinical conditions such as mood disorders and cognitive dysfunction (defined as a significant defect in attention, memory, language, reasoning, execution, visual-spatial processing, and psychomotor speed^409^), represent an important type of SLE comorbidities. Among these varied disease manifestations, cognitive dysfunction has the highest prevalence, with up to 50% SLE patients carrying overt neurological syndromes being accompanied with varied degrees of cognitive dysfunctions.^410^ The overall prevalence of cognitive dysfunction varies between 3% and 88% among SLE patients^411–414^ due to the lack of consensus in screening tools and validated markers for identifying cognitive dysfunctions, difficulty in associating cognitive dysfunctions with SLE, and heterogeneity regarding the severity and cohort intrinsic feature of SLE patients.^410,415–419^ Clinically, only 3-5% SLE patients carry severe cognitive dysfunctions, and the degrees of the cognitive syndromes of most patients are mild-to-moderate following a benign course.^420^

Conventional neuroimaging have shown that, although varying significantly among individuals, cognitive dysfunctions primarily display periventricular hyperintensities and cerebral atrophy.^421^ The pathogenesis of SLE-related cognitive dysfunctions are considered to be caused by the access of peripherally produced neurotoxic auto-antibodies to the central nervous system, the process of which involves the breach of the blood-brain barrier (BBB).^422^ Therefore, neuropsychiatric disorders can be roughly considered as a manifestation of cardiovascular syndromes commonly occurred among SLE carriers in the brain system, leaving risk factors predisposing the development of neuropsychiatric and cardiovascular diseases associated with SLE similar. Besides physiological factors that are difficult to manage by nurture, the negative impacts of some lifestyles and medical predisposing factors of cardiovascular comorbidities should be removed to keep the mental health of SLE carriers. These include the avoidance of smoking^423^ and abused use of glucocorticoids as aforementioned. Of note is that both long- and short- term application of glucocorticoids for SLE control have been shown capable of worsening cognitive dysfunction among SLE patients by several clinical studies.^424^ For instance, long-term use of moderate doses of prednisolone led to reduced cognitive flexibility and decision-making ability of SLE patients;^425^ a daily use of prednisolone for over 9 mg worsened the mathematical processing ability of SLE patients with SLE;^426^ short-term application of high-dose glucocorticoid resulted in hippocampal atrophy and declarative memory defects.^427^ The mechanistic impact of glucocorticoids on the cognitive system of SLE patients can be attributed to the interactions of these agents with the neuronal receptors distributed in the prefrontal cortex, hippocampus and basolateral amygdala, which lead to impaired memory and learning activities.^428,429^ On the other hand, several observational studies have supported the utility of aspirin in rescuing cognitive dysfunctions.^430–435^ For instance, regular administration of low-dose aspirin improved the cognitive function of the 123 SLE patients recruited from a 3-year prospective study, with a particular beneficial impact observed among the elder individuals also possessing cardiovascular risk factors such as diabetes.^430^ However, no consensus has been reached regarding the use of statin^436^ for treating cognitive dysfunctions according to current clinical settings as has been recommended for preventing cardiovascular comorbidities.

In addition, individualized multipronged strategies specifically designed for mental health management have been recommended for preventing cognitive dysfunctions. These can be categorized into non-pharmacological and pharmacological approaches.

Non-pharmacological interventions include the engagement of regular physical activities and cognitive rehabilitation.^422^ Regular physical activities encompass three lifestyle components, i.e., mental, physical, social,^437–439^ which regain the cognitive functionalities via, e.g., improving neurovascular coupling that is defined as increased neuronal activities linked to the local regulation of cerebral blood flow.^440^ The benefits of physical activities to cognitive behavior improvement is dose-dependent. That is, the risk of developing cognitive deterioration can be ameliorated by taking moderate-intensity aerobic exercises for 30 min and 5 days per week or, alternatively, by taking high-intensity aerobic exercises for above 20 min and 3 days per week.^441^ Cognitive rehabilitation is performed by occupational therapists to intensively retrain the cognitive and memory skills of the patients,^442,443^ the process of which is comprised of, but not limited to, cognitive behavior intervention, cognitive training exercises, prioritization, time optimization and memory aids.^444^ Cognitive behavior intervention aims at helping SLE patients with self-perceived cognitive dysfunctions to improve their memories and the abilities to perform daily activities.^445^ Cognitive training exercises such as chess can help the patients enhance their executive functions and problem-resolving skills.^444^ Prioritization can help SLE patients carrying cognitive dysfunctions to focus on one task prior to proceeding to the next. Time optimization helps the patients to prioritize cognitively intensive tasks to the earlier time of a day. Memory aids aim to help the patients to better manage their daily activities that can be in the form of, e.g., written reminders.^422^

Pharmacological medications used for improving cognitive dysfunctions include, primarily, N-methyl-D-aspartate receptor (NMDAR) antagonists, acetylcholinesterase inhibitors, and C5a receptor blocking agents.

The NMDAR is an ionotropic glutamate receptor mediating excitatory neurotransmission in the mammalian brain, which are localized in the postsynaptic terminal allowing for the influx of sodium (Na^+^) and Ca^2+^. NMDAR plays essential roles during synaptic transmission for synaptic plasticity and normal neuronal activities. Thus, NMDAR antagonists such as memantine can help the patients regain memory and learning abilities, and thus have been considered for managing patients carrying moderate-to-severe dementia.^446^

Acetylcholinesterase is an enzyme residing largely in the synaptic cleft that rapidly breaks down acetylcholine molecules to prevent prolonged action and allow for their in time recycling. Acetylcholinesterase inhibitors such as donepezil, rivastigmine, galantamine thus can help improve the cognitive abilities of the patients by increasing the availability of acetylcholines at the synaptic clefts and avoiding their overt activities.^447^ According to a meta-analysis involving 10 randomized, double-blind, placebo-controlled clinical trials of a 6-month treatment of acetylcholinesterase inhibitors, these agents were found to be associated with reduced cognition decline.^448,449^

The BBB is an unique structure in the brain for the maintenance of brain homeostasis. The complement activation byproduct C5a can elevate the permeability of BBB by relaying signals through its G-protein coupled receptor C5aR1. Thus, C5a receptor blockage therapeutics can prevent SLE patients from developing cognitive syndromes by keeping the integrity of BBB.^450^ It has been shown using MRL/lpr lupus prone mice that C5a receptor blockade can effectively ameliorate BBB disruption for attenuated cognitive abnormalities.^451^

## Current therapeutics for SLE treatment

### Current therapeutics attenuating immune response

TLR signaling plays a central role in the development of SLE. Endosomal TLRs (including TLR3, TLR7, TLR8, TLR9, and TLR13) that are specialized to sense nucleic acids detect self-derived damage-associated molecular patterns (DAMPs) together with surface TLRs to collectively trigger the downstream production of pro-inflammatory cytokines. Therefore, disturbing TLR signaling remains a strategy to fight against SLE. Enpatoran, being a highly selective dual TLR7 and TLR8 inhibitor, has been developed for treating autoimmune disorders including SLE, CLE and myositis. Several clinical trials including the ethno-bridging phase I trial (NCT04880213) and the ongoing phase II trials (NCT05162586, NCT05540327) have been or being performed to examine the treatment efficacy of enpatoran, as well as its pharmacokinetic, pharmacodynamic, and clinical safety.^452^ Current clinical and pre-clinical evidence have implicated a potential glucocorticoid-sparing effect of enpatoran for SLE treatment and the promoted overall health among SLE patients receiving enpatoran.^452,453^ E6742, another selective dual TLR7 and TLR8 inhibitor was found capable of ameliorating the pathogenesis features of lupus mice,^454^ yet its clinical evidence from a phase I/II clinical trial (NCT05278663), though been completed, has not been published. On the other hand, anti-malarial agents such as hydroxychloroquine, chloroquine, quinacrine and artemisinin can impede TLR from recognizing nucleic acids via masking TLR-binding epitopes of nucleic acids and thus be feasible for SLE treatment as well.^455–462^ While hydroxychloroquine has been recommended for all SLE patients due to its comparatively lower incidence of developing side effects such as retinopathy and cardiomyopathy than other antimalarial agents via inhibiting TLR3/7/9,^348,463^ chloroquine resolved skin lesions among CLE patients by functioning as a TLR7/8/9 antagonist,^459^ quinacrine ameliorated skin conditions of CLE patients through targeting TLR7/9,^462^ and artemisinin prevented the recurrence of LN via inhibiting TLR4 (Table 2).^460^

**Table 2:** Medications available for SLE treatment approved or under clinical investigations

Commercial name	Medication type	Trial number (phase, status)	Mechanism of action	Note	References
Enpatoran	TLR7/8 inhibitor	NCT05162586 (II, ongoing), NCT05540327 (II, ongoing)	Attenuating immune response	Suppressing TLR signaling	^453^
E6742	TLR7/8 inhibitor	NCT05278663 (I/II, completed)	Attenuating immune response	Suppressing TLR signaling	^454^
Hydroxychloroquine	Anti-malaril TLR7/3/9 atagonist	In clinics	Attenuating immune response	Suppressing TLR signaling	^506^
Chloroquine	Anti-malarial TLR7/8/9 atagonist	In clinics	Attenuating immune response	Suppressing TLR signaling	^459^
Quinacrine	Anti-malaril TLR7/9 atagonist	In clinics	Attenuating immune response	Suppressing TLR signaling	^461^
Artemisinin	Anti-malaril TLR4 atagonist	In clinics	Attenuating immune response	Suppressing TLR signaling	^460^
Litifilimab	Anti-CD303 antibody	NCT05352919 (III, ongoing), NCT04961567 (III, ongoing), NCT04895241 (III, ongoing), NCT06044337 (III, ongoing), NCT05531565 (II/III, ongoing), NCT02847598 (II, completed), NCT02106897 (I, completed)	Attenuating immune response	Suppressing TLR signaling	^467,468^
Anifrolumab	IFNα receptor antibody	In clinics	Attenuating immune response	Suppressing TLR signaling	^472^
IFNα kinoid	IFNα vaccine	NCT02665364 (I/II, completed), NCT01058343 (I/II, completed)	Attenuating immune response	Suppressing TLR signaling	^108,475^
Rontalizumab	IFNα antibody	NCT00962832 (II, completed)	Attenuating immune response	Suppressing TLR signaling	^477^
Sifalimumab	IFNα antibody	NCT00979654 (II, completed), NCT01283139 (IIb, completed), NCT00657189 (II, completed), NCT00299819 (I, completed), NCT01031836 (II, completed), NCT00482989 (I, completed)	Attenuating immune response	Suppressing TLR signaling	^476,477^
Upadacitinib	JAK1 inhibitor	NCT05843643 (III, ongoing)	Attenuating immune response	Suppressing JAK/STAT signaling	^110^
Filgotinib	JAK1 inhibitor	NCT03978520 (II, completed), NCT03134222 (II, completed),	Attenuating immune response	Suppressing JAK/STAT signaling	^478,479^
Fludarabine	STAT1 inhibitor	NCT00001676 (I, completed)	Attenuating immune response	Suppressing JAK/STAT signaling	^481^
Artesunate	STAT3 inhibitor	NCT03214731 (IV, NA)	Attenuating immune response	Suppressing JAK/STAT signaling	^482^
Azathioprine	Purine antagonist	In clinics	Attenuating immune response	Immunosuppressor	^492,499^
Cyclophosphamide	Alkylating agent	In clinics	Attenuating immune response	Immunosuppressor	^481,494,497^
Mycophenolate mofetil	IMPDH inhibitor	In clinics	Attenuating immune response	Immunosuppressor	^499–501,507,510,511^
Mizoribine	IMPDH inhibitor	NCT02256150 (III, completed)	Attenuating immune response	Immunosuppressor	^490,502^
Leflunomide	DHOH inhibitor	In clinics	Attenuating immune response	Immunosuppressor	^492,494,504^
Cyclosporine A	Calcineurin inhibitor	In clinics	Attenuating immune response	Immunosuppressor	^493,496^
Tacrolimus	Calcineurin inhibitor	In clinics	Attenuating immune response	Immunosuppressor	^509–511^
Voclosporin	Calcineurin inhibitor	NCT02949973 (II, completed), NCT05288855 (III, ongoing), NCT03021499 (III, completed), NCT02141672 (II, completed), NCT03597464 (III, completed), NCT06406205 (III, ongoing), NCT05306873 (II, ongoing)	Attenuating immune response	Immunosuppressor	^512,513,515,516^
Autoreactive T cell vaccine	Autoreactive T cell vaccine	Clinical case study	Restoring cytokine microenvironment homeostasis	Targeting T cells	^599^
Rozibafusp alfa	ICOSL and BLyS inhibitotr	NCT02618967 (II, completed), NCT04058028 (II, completed)	Attenuating immune response	Targeting B cells	^535^
Dapirolizumab Pegol	CD40L antibody	NCT04976322 (III, ongoing), NCT02804763 (II, completed), NCT04294667 (III, completed)	Attenuating immune response	Targeting B cells	^519^
Rigerimod	small nuclear U1RNP-70K derived peptide	NCT02504645 (II, completed)	Attenuating immune response	Targeting B cells	^486^
Belimumab	BLyS inhibitor	In clinics	Attenuating immune response	Targeting B cells	^522,523^
Blisibimod	BLyS inhibitor	NCT01395745 (III, completed), NCT02514967 (III, terminated), NCT02074020 (III, withdrawn), NCT01305746 (II, completed), NCT01162681 (II, completed)	Attenuating immune response	Targeting B cells	^525,526^
Tabalumab	BLyS inhibitor	NCT02041091 (III, terminated), NCT01488708 (III, terminated), NCT01205438 (III, completed), NCT01196091 (III, completed)	Attenuating immune response	Targeting B cells	^527,528^
Ianalumab	BLyS-R inhibitor	NCT06411639 (I, ongoing), NCT06133972 (III, ongoing), NCT06293365 (II, ongoing), NCT05126277 (III, ongoing), NCT05639114 (III, ongoing), NCT05624749 (III, ongoing), NCT03656562 (II, ongoing)	Attenuating immune response	Targeting B cells	^529^
Telitacicept	BLyS and APRIL inhibitor	In clinics	Attenuating immune response	Targeting B cells	^533^
Atacicept	BLyS and APRIL inhibitor	NCT02070978 (II, terminated), NCT01972568 (II, completed), NCT00624338 (II/III, completed), NCT00573157 (II/III, terminated)	Attenuating immune response	Targeting B cells	^531,532^
Elsubrutinib+Upadacitinib	BTK inhibitor+JAK1 inhibitor	NCT03978520 (II, completed), NCT04451772 (II, completed)	Attenuating immune response	Targeting B cells	^110^
Elsubrutinib	BTK inhibitor	NCT04451772 (II, completed), NCT03978520 (II, completed)	Attenuating immune response	Targeting B cells	^110^
Branebrutinib	BTK inhibitor	NCT04186871 (II, completed)	Attenuating immune response	Targeting B cells	^539^
Fenebrutinib	BTK inhibitor	NCT02908100 (II, completed)	Attenuating immune response	Targeting B cells	^540^
Evobrutinib	BTK inhibitor	NCT02975336 (II, terminated), NCT02537028 (I, completed)	Attenuating immune response	Targeting B cells	^541^
Orelabrutinib	BTK inhibitor	NCT04305197 (I/II, completed), NCT05688696 (II, ongoing)	Attenuating immune response	Targeting B cells	^542,543^
Zanubrutinib	BTK inhibitor	NCT04643470 (II, ongoing)	Attenuating immune response	Targeting B cells	^544^
AC0058TA	BTK inhibitor	NCT03878303 (I, NA)	Attenuating immune response	Targeting B cells	^545^
Obexelimab	CD19 and FcγRIIb antibody	NCT02725515 (II, completed) NCT06559163(II, ongoing)	Attenuating immune response	Targeting B cells	^548^
autologous CD19 CAR-T cells	CD19 CAR-T cells	NCT06150651 (I, ongoing), NCT06316791 (I, ongoing), NCT06333483 (I, ongoing), NCT06342960 (I/II, ongoing), NCT05869955 (I, ongoing), NCT06316076 (I, ongoing) NCT06121297 (I/II, ongoing), NCT06189157 (I/II, ongoing), NCT06316791 (I, ongoing), NCT06347718 (I/II, ongoing), NCT05938725 (I/II, ongoing), NCT03030976 (I, NA)	Attenuating immune response	Targeting B cells	^551,552^
autologous CD19/BCMA CAR-T cells	CD19/BCMA CAR-T cells	NCT06428188 (I/II, ongoing), NCT05846347 (I, ongoing), NCT06503224 (NA, ongoing), NCT06530849 (I/II, ongoing), NCT06285279 (I, ongoing)	Attenuating immune response	Targeting B cells	^555^
autologous BCMA CAR-T cells	BCMA CAR-T cells	NCT06038474 (II, ongoing)	Attenuating immune response	Targeting B cells	^556^
autologous CD20/BCMA CAR-T cells	CD20/BCMA CAR-T cells	NCT06249438(I, ongoing)	Attenuating immune response	Targeting B cells	^557^
autologous CD19/CD20 CAR-T cells	CD19/CD20 CAR-T cells	NCT06462144 (I, ongoing), NCT06153095 (I/II, ongoing), NCT06567080 (I, ongoing)	Attenuating immune response	Targeting B cells	^558–560^
allogeneic CD19 CAR-T cells	allogeneic CD19 CAR-T cells	NCT05988216 (NA, ongoing), NCT06340490 (I, ongoing), NCT05859997 (NA, ongoing), NCT06429800 (I, NA), NCT06294236 (I, ongoing), NCT06375993 (I, NA)	Attenuating immune response	Targeting B cells	^561–566^
CD19 CAR-NK cells	CD19 CAR-NK cells	NCT06421701 (I, ongoing), NCT06010472 (I, ongoing), NCT06557265 (I, ongoing), NCT06518668 (I, ongoing), NCT06468683 (I, NA), NCT06377228 (I, NA), NCT06255028 (I, ongoing)	Attenuating immune response	Targeting B cells	^567,568^
Rituximab	CD20 antibody	NCT00036491 (I/II, completed), NCT00556192 (II, completed), NCT03312907 (III, completed), NCT02284984 (II, completed), NCT00381810 (III, terminated), NCT00137969 (II/III, completed), NCT00282347 (III, completed), NCT00293072 (II, completed),NCT05207358 (IV, ongoing), NCT05828147 (IV, ongoing), NCT04127747 (IV, NA)	Attenuating immune response	Targeting B cells	^495,570,571^
Rituximab+AB-101	CD20 antibody+NK cells	NCT06265220 (I, ongoing), NCT06581562 (I, ongoing)	Attenuating immune response	Targeting B cells	^574,575^
Ofatumumab	CD20 antibody	Clinical case study	Attenuating immune response	Targeting B cells	^576^
Ocrelizumab	CD20 antibody	NCT00626197 (III, terminated), NCT00539838 (III, terminated)	Attenuating immune response	Targeting B cells	^577^
Obinutuzumab	CD20 antibody	NCT05039619 (II, ongoing), NCT02550652 (II, completed), NCT04702256 (III, ongoing), NCT04221477 (III, ongoing), NCT04963296 (III, ongoing)	Attenuating immune response	Targeting B cells	^578^
Epratuzumab	CD22 antibody	NCT01408576 (III, completed), NCT01262365 (III, completed), NCT01261793 (III, completed), NCT01534403 (II, completed), NCT01449071 (I/II, completed), NCT02306629 (I, completed), NCT00660881 (II, completed), NCT00383513 (II, completed), NCT00624351 (II, completed), NCT00011908 (I, completed), NCT00383214 (III, terminatied), NCT00111306 (III, terminatied)	Attenuating immune response	Targeting B cells	^102,106^
Abatacept	Fusion protein interrupting CD80/CD86 signaling	NCT00705367 (I, completed), NCT01714817 (III, terminated), NCT00119678 (II, completed), NCT00774852 (II, completed), NCT02270957 (II, completed), NCT00430677 (II/III, terminated), NCT02429934 (II/III, terminated), NCT04186871 (II, completed)	Attenuating immune response	Targeting B cells	^583,585^
Daratumumab	CD38 antibody	NCT04868838 (II, ongoing), NCT04810754 (II, NA)	Attenuating immune response	Targeting B cells	^588,709]^
Bortezomib	Proteasome inhibitor	NCT01169857 (IV, withdrawn), NCT02102594 (II, terminated)	Attenuating immune response	Targeting B cells	^589,591^
Abetimus sodium	Crosslinking dsDNA receptor on B cells	NCT00390091 (II, withdrawn), NCT00035308 (III, completed), NCT00089804 (III, terminated)	Attenuating immune response	Targeting B cells	^601^
Immunoglobulin	Immunoglobulin	NCT01841619 (I, completed), NCT00460928 (I, completed)	Attenuating immune response	Targeting B cells	^602^
Eculizumab	C5 antibody	Clinical case study	Attenuating immune response	Attenuating over-activated complement system	^604–606^
Ravulizumab	C5 antibody	NCT04564339 (II, ongoing)	Attenuating immune response	Attenuating over-activated complement system	^607^
Avacopan	C5a receptor inhibitor	NCT05984251 (I, completed)	Attenuating immune response	Attenuating over-activated complement system	^608^
Pegcetacoplan	C3 inhibitor	NCT03453619 (II, completed)	Attenuating immune response	Attenuating over-activated complement system	^609^
Tocilizumab	IL6 receptor antibody	NCT05835986 (I, ongoing), NCT05155345 (I, ongoing)	Restoring cytokine microenvironment homeostasis	Th1/Th2 balance	^612^
PF-04236921	IL6 antibody	NCT01405196 (II, completed)	Restoring cytokine microenvironment homeostasis	Th1/Th2 balance	^107^
Sirukumab	IL6 antibody	NCT01273389 (II, completed), NCT01702740 (I, completed)	Restoring cytokine microenvironment homeostasis	Th1/Th2 balance	^614^
Ustekinumab	IL12 and IL23 antibody	NCT02349061 (II, completed), NCT04060888 (III, withdrawn), NCT03517722 (II, terminated)	Restoring cytokine microenvironment homeostasis	Th1/Th2 balance	^616,617^
Apremilast	PDE4 inhibitor	NCT00708916 (I/II, completed)	Restoring cytokine microenvironment homeostasis	Th1/Th2 balance, Treg/Th17 balance	^619^
Low dose IL2	Low dose IL2	NCT04077684 (II, ongoing), NCT05339217 (III, ongoing), NCT05631717 (III, ongoing), NCT03312335 (II, completed), NCT01988506 (II, completed), NCT05262686 (III, NA), NCT02084238 (NA, completed), NCT02932137 (NA, completed), NCT04397107 (NA, completed)	Restoring cytokine microenvironment homeostasis	Treg/Th17 balance	^628,629,631^
Stem cells	Stem cells	NCT00076752 (II, completed), NCT03917797 (II, ongoing), NCT03673748 (II, ongoing), NCT04318600 (I, completed), NCT04184258 (I/II, completed), NCT03171194 (I, completed), NCT02633163 (II, ongoing), NCT05018858 (I, ongoing), NCT00271934 (II, completed), NCT03828071 (NA, completed)	Restoring cytokine microenvironment homeostasis	Treg/Th17 balance	^622–624^
BT063	IL-10 antibody	NCT02554019 (II, completed)	Restoring cytokine microenvironment homeostasis	Treg/Th17 balance	^632^
Secukinumab	IL17A antibody	NCT05232864 (III, terminated), NCT04181762 (III, terminated), NCT03866317 (II, withdrawn)	Restoring cytokine microenvironment homeostasis	Treg/Th17 balance	^635–637^
Exosomes from MSCs	Exosomes from MSC	Clinical case study	Restoring cytokine microenvironment homeostasis	Treg/Th17 balance	^638^
Betamethasone	Glucocorticoid	In clinics	Restoring cytokine microenvironment homeostasis	Treg/Th17 balance	^639^
Dexamethasone	Glucocorticoid	In clinics	Restoring cytokine microenvironment homeostasis	Treg/Th17 balance	^640^
Hydrocortisone	Glucocorticoid	In clinics	Restoring cytokine microenvironment homeostasis	Treg/Th17 balance	^641^
Prednisone	Glucocorticoid	In clinics	Restoring cytokine microenvironment homeostasis	Treg/Th17 balance	^642^
Prednisolone	Glucocorticoid	In clinics	Restoring cytokine microenvironment homeostasis	Treg/Th17 balance	^643^
Triamcinolone	Glucocorticoid	In clinics	Restoring cytokine microenvironment homeostasis	Treg/Th17 balance	^644,645^
Methylprednisolone	Glucocorticoid	In clinics	Restoring cytokine microenvironment homeostasis	Treg/Th17 balance	^647^
Rapamycin	mTOR inhibitor	NCT00779194 (I/II completed), NCT04582136 (II, ongoing), NCT04892212 (I/II, NA), NCT00392951 (I/II, completed), NCT0473695 (II, planned)	Restoring cytokine microenvironment homeostasis	Treg/Th17 balance	^654,655^
Atorvastatin	Statin	NCT00065806 (III, completed)	Restoring cytokine microenvironment homeostasis	Treg/Th17 balance	^657^
Pravastatin	Statin	NCT00054938 (II, completed)	Restoring cytokine microenvironment homeostasis	Treg/Th17 balance	^659^
Rosuvastatin	Statin	NCT01170585 (II, completed)	Restoring cytokine microenvironment homeostasis	Treg/Th17 balance	^660^
Simvastatin	Statin	NCT01953835 (I, completed)	Restoring cytokine microenvironment homeostasis	Treg/Th17 balance	^661^
Plasma exchange	Complement	Clinical case study	Rescuing impaired debris clearance machinery	Adding complements Decrease ICs	^667–669^
Plasma transfusion	Complement	Clinical case study	Rescuing impaired debris clearance machinery	Adding complements	^665,666^
Valziflocept	soluble human FcγRIIb	clinical trial (ACR Meeting Abstracts)	Rescuing impaired debris clearance machinery	Decrease ICs deposition	^663,664^

*APRIL* a proliferation-inducing ligand, *BCMA* B cell maturation antigen, *BLyS* B lymphocyte stimulator, *BTK* Bruton’s tyrosine kinase, *CAR* chimeric antigen receptor, *DHOH* dihydroorotate dehydrogenase, *IFN* interferon, *IMPDH* inosine monophosphate dehydrogenase, *JAK* Janus kinase, *STAT* signal transducer and activator of transcription, *TLR* toll-like receptor, *TNF* tumor necrosis factor

Strategies targeting type I IFNs for attenuated autoimmune response and SLE severity have been established for SLE treatment. For instance, litifilimab, an antibody against blood DC antigen 2 (BDCA2, also named CD303), which is expressed exclusively on plasmacytoid DCs^464^ and a major predictor of type I IFNs production,^465^ decreased the expression of genes relaying IFN signals among SLE patients in a phase I clinical study (NCT02106897)^466^ and attenuated the activities of SLE and CLE in a phase II clinical trial (NCT02847598).^467,468^ Anifrolumab, a human monoclonal antibody against type I IFNs receptor subunit 1 exhibited improved complete renal response among LN patients and reduced glucocorticoid use in a phase II randomized trial (NCT02547922),^469^ elevated the response rate from a phase III trial (NCT02446899),^103^ and long-term safety and tolerability in a randomized placebo-controlled phase III extension trial (NCT02794285).^470^ Combining the standard therapy with anifrolumab showed higher remission rates than using the standard therapy alone in SLE management according to a 4-year study.^471^ Attributing to these clinical successes, anifrolumab received its first approval as an add-on therapy for treating moderate-to-severe SLE in the United States in July 2021,^472^ in Japan in September 2021, in the Europe in February 2022, in Hong Kong in December 2022, and in the Guangdong province of China in October 2024.^473,474^ Medications directly targeting IFNα have also demonstrated positive results on SLE management. For instance, IFNα kinoid, a vaccine inducing neutralizing antibodies against IFNα, significantly reduced the IFN gene signature and attenuated symptoms with an acceptable safety profile in a phase IIb study (NCT02665364)^108^ and phase I/II trial (NCT01058343) among SLE patients.^475^ Anti-IFNα antibodies such as rontalizumab and sifalimumab showed reduced flares, decreased steroid use and improvement management of SLE patients in a phase II (NCT00962832) and phase IIb (NCT01283139) clinical trial, respectively^476,477^ (Table 2).

Strategies targeting the JAK/STAT axis have also been shown feasible for treating SLE via intervening type I IFN mediated signaling. These include, e.g., JAK inhibitors such as upadacitinib and filgotinib, as well as STAT inhibitors such as fludarabine and artesunate. Specifically, upadacitinib reduced the flares of SLE patients with good tolerance in a phase II clinical trial (NCT03978520);^110^ and filgotinib showed desirable therapeutic efficacy in treating lupus membranous nephropathy according to a phase II clinical trial (NCT03285711)^478^ and in treating moderate-to-severe CLE based on results from another phase II study (NCT03134222).^479^ Combined use of low-dose fludarabine and cyclophosphamide for a short duration led to long-lasting disease remission among LN patients but bone marrow toxicity from a phase I/II study;^480,481^ and artesunate demonstrated its therapeutic efficacy and safety in treating active lupus nephritis in a phase IV trial (NCT03214731) when being coupled with the standard of care^482^ (Table 2).

Strategies impairing the presentation of antigens to T cells by innate lymphocyte cells (ILCs) have also been established for SLE treatment. For example, rigerimod (a 21-mer linear peptide derived from the small nuclear U1RNP-70K) inhibits B cell maturation by reducing the stability of MHC II molecules towards blocked antigen presentation to auto-reactive T cells.^483,484^ Rigerimod improved the clinical symptoms of 20 moderate SLE patients,^485^ and slightly outweighed placebo in a phase III trial examining its clinical efficacy in treating SLE (NCT02504645).^486^

Immunosuppressors such as mycophenolate mofetil, azathioprine, cyclophosphamide, mizoribine, and leflunomide have been used for SLE treatment, attributing to their roles in imposing cytotoxic effects on rapidly growing cells including proliferating T and B lymphocytes.^487–490^ Specifically, mycophenolate mofetil obstructs the formation of guanine nucleotides via inhibiting inosine monophosphate dehydrogenase (IMPDH) and thus disturbs the generation of DNA necessary for cell replication;^489^ azathioprine is a purine antagonist blocking nucleotide synthesis and inhibiting leukocyte proliferation;^488^ and cyclophosphamide functions as an alkylating agent disrupting DNA replication.^487^ Mycophenolate mofetil, azathioprine and cyclophosphamide have all been widely used in the clinics for SLE management with considerable good therapeutic responses received. Yet, cyclophosphamide is more inclined to be used as an induction therapy (often applied as the first-phase therapeutics) for treating severe conditions, azathioprine is prone to maintain the disease situations, and mycophenolate mofetil serves both therapeutic purposes^481,491–501^ with comparable efficacy and reduced toxicity.^500^ Mizoribine, functioning similarly with mycophenolate mofetil, enhanced the clinical and serological indexes of 5 SLE patients.^502^ Leflunomide works as a dihydroorotate dehydrogenase (DHOH) inhibitor to interfere with pyrimidine synthesis.^503^ It has been shown that low-dose leflunomide effectively improved the treatment efficacy and safety of Chinese LE patients receiving prednisone therapeutics,^494^ and a trial involving 17 LE patients demonstrated the safety and efficacy of leflunomide in treating refractory LE patients or those having developed resistance to conventional therapies.^504^ Leflunomide displayed a similar efficacy with azathioprine in treating LN (NCT01172002).^492^ In addition, calcineurin inhibitors such as cyclosporine A, tacrolimus and voclosporin have been used for SLE management via executing their immunosuppressive functions.^505,506^ For instance, cyclosporine A improved the therapeutic outcome of LN receiving prednisolone (a type of corticosteroids);^493^ and was shown as effective as cyclophosphamide in treating LN patients according to a phase II trial (NCT00976300).^496^ Tacrolimus suppressed T cell activation and B cell differentiation by inhibiting the calcineurin pathway as a result of inhibited dephosphorylation and translocation of NFAT.^507,508^ A prospective clinical case study involving 19 patients demonstrated the feasibility and safety of long-term and low-dose use of tacrolimus for treating young LN patients;^509^ and a phase IV clinical trial composed of 150 LN patients and an unknown phase study reported comparable treatment efficacies between tacrolimus and mycophenolate mofetil in the long run (NCT00371319).^510,511^ Voclosporin, another calcineurin inhibitor,^512,513^ showed superior safety, efficacy and feasibility for its long-term use in phase III clinical trials against LN (NCT03021499, NCT01580865, NCT03597464).^511,514,515^ A phase II clinical trial demonstrated that low-dose voclosporin could serve as the induction therapy for treating active LN for enhanced renal response rate (NCT02141672) (Table 2).^516^

The pair of CD40 receptor and its ligand CD40L (i.e., CD40-CD40L) is one of the most critical molecular axes mediating the crosstalk between T and B cells.^517^ While B cells constitutively express high levels of CD40, T cells express high levels of CD40L upon activation.^517^ Due to the essential role of the CD40-CD40L interaction played during the adaptive immune response, agents targeting CD40 or CD40L have been established that had held a great promise. For instance, dapirolizumab pegol has demonstrated its efficacy and safety in treating SLE via targeting CD40L according to a phase II trial (NCT02804763),^518,519^ and is currently undergoing two phase III trials as a therapeutic against SLE (NCT04976322, NCT04294667) (Table 2).

BLyS and a proliferation-inducing ligand (APRIL) are vital survival factors regulating B cell survival, proliferation and differentiation.^520^ Thus, BLyS inhibitors may execute immunosuppression capacity via dampening the activation and proliferation of B cells.^521^ Belimumab, an inhibitor of BLyS having been approved by FDA for SLE management in 2011, has been effective in dealing with and preventing renal damages among SLE patients.^522–524^ Blisibimod, another BLyS inhibitor, has been associated with reduced steroids, decreased proteinuria among SLE patients in a phase III study (NCT01395745);^525^ blisibimod also significantly reduced levels of proteinuria, anti-dsDNA antibody, B cells but increased the amount of complements C3 and C4 in a phase II study (NCT01162681).^526^ Tabalumab, through suppressing BLyS, displayed positive therapeutic changes regarding the levels of anti-dsDNA antibody, complements, B cells and immunoglobulins according to two phase III studies (NCT01196091, NCT01205438).^527,528^ Several clinical trials are being conducted to investigate the efficacy of ianalumab in treating SLE as an inhibitor of the BLyS receptor.^529^ Atacicept, a recombinant fusion protein concomitantly blocking BLyS and APRIL, showed therapeutic fitness in treating SLE patients especially for those with high disease activity in a phase II study (NCT01972568).^530^ Atacicept was also associated with reduced levels of total IgG and anti-dsDNA antibody as well as increased amount of C3 and C4 in a phase II/III study (NCT00624338).^531,532^ Telitacicept is a fusion protein comprised of a recombinant transmembrane activator and calcium modulator and cyclophilin ligand interactor (TACI) receptor fused to the fragment crystallizable (Fc) domain of human IgG. By binding to and neutralizing the activity of BLyS and APRIL, telitacicept suppresses the development and survival of PCs and mature B cells. Telitacicept received its first approval in China for treating active SLE patients in 2021.^533^ Rozibafusp alfa (AMG 570) is a first-in-class bispecific IgG2-peptide fusion protein designed to target both BLyS and inducible T-cell costimulator ligand (ICOSL), where ICOSL is mainly expressed on B cells and APCs. By suppressing BLyS, rozibafusp alfa dampens the differentiation and survival of B cells and PCs. In addition, rozibafusp alfa blocks the interactions between Th cells (especially Tfh) and B cells for impaired antibody production as a result of inhibited ICOSL. The therapeutic efficacy of rozibafusp alfa was promising according to the results obtained from mouse SLE models.^534^ Phase I and II clinical trials examining the feasibility of using rozibafusp alfa for SLE treatment have been launched with the results not available yet (NCT02618967, NCT04058028).^535^ (Table 2).

Bruton’s tyrosine kinase (BTK), an intracellular signaling molecule of B and myeloid cell pathways, is of the vital importance for B cell development and activities. Activated BTK signaling is associated with elevated production of auto-antibodies that can form ICs with auto-antigens and deposit in tissues to induce inflammation and damage the lesions.^536,537^ BTK inhibitors such as elsubrutinib, branebrutinib, fenebrutinib and evobrutinib, thus, represent a portfolio of promising immunosuppressors coping with SLE. Elsubrutinib, in combination with upadacitinib (a JAK1 inhibitor), improved SLE clinical syndromes without obvious adverse concerns in a phase II study (NCT03978520).^110^ Branebrutinib, another BTK inhibitor with demonstrated in vivo evidence showing its superiority in treating SLE,^538^ is currently under clinical investigation (NCT04186871).^539^ The BTK inhibitor fenebrutinib reduced the levels of anti-dsDNA antibody and total IgG, and increased that of complement C4 among SLE patients according to a phase II study (NCT02908100).^540^ Evobrutinib displayed a positive long-term efficacy in treating SLE patients carrying relapsed multiple sclerosis in a phase II trial (NCT02975336).^541^ More BTK inhibitors are under clinical investigations regarding their fitness in treating that, include, e.g., orelabrutinib (NCT04305197, NCT05688696),^542,543^ zanubrutinib (NCT04643470),^544^ and AC0058TA (NCT03878303) (Table 2).^545^

Besides inhibiting B cell activation, B cell depletion represents another portfolio of strategies attenuating over-activated adaptive immune response for SLE control. CD19 and CD20 are B cell linkage-specific antigens expressed on the surface of most B cell lymphocytes, with CD19 being expressed across the entire spectrum during B cell maturation and CD20 being present during the late stages of B cell lymphogenesis.^546,547^ Obexelimab is a monoclonal antibody binding to CD19 and FcyRIIb, and has been reported by a double-blind, randomized, placebo-controlled phase II study capable of reducing the amount of B cells in SLE patients for improved therapeutic response and efficacy (NCT02725515).^548^ Therapeutics utilizing chimeric antigen receptor (CAR)-T cells to target CD19 and/or B cell maturation antigen (BCMA) expressed on B cells or their end-stage PCs have proven to be a revolutionary successful regimen for treating diseases associated with over-activated B cells including SLE.^549^ Mechanically, hyperactive B cells can cause inflammation and tissue damage by producing overt autoantibodies, and carefully designed CAR-T therapeutics can reset the immune system to alleviate the disease symptoms.^550,551^ For example, CAR-T cells targeting CD19 hold a significant promise in treating refractory SLE patients, with rapid and profound depletion of B cells and concomitant improvement in clinical symptoms and serological markers being reported.^551–555^ A phase I study employing a dual targeting strategy against CD19 and BCMA depleted both B cells and PCs, with the majority of the SLE patients showing negative results for all autoantibodies including those secreted by long-lived PCs after the treatment.^555^ More clinical trials on CAR-T cells targeting other B cell specific antigens or antigen combinations such as BCMA CAR-T cells (NCT06038474),^556^ CD20/BCMA CAR-T cells (NCT06249438),^557^ and CD19/CD20 CAR-T cells (NCT06462144, NCT06153095, NCT06567080) in treating SLE have been registered.^558–560^ Despite these encouraging clinical results regarding the safety and efficacy of CAR-T cell therapeutics in treating SLE patients, challenges exist that have substantially hindered the wide adoption of this promising approach for SLE management. First, the long-term persistence and functionality of CAR-based immune cells are not completely understood, and there is a need for ongoing monitoring to assess the durability of therapeutic response and the potential of disease relapse.^549^ Second, the risk of developing cytokine release syndrome (CRS) and immune effector cell-associated neurotoxicity syndrome (ICANS) remain a concern, although it has been considered to be milder in the autoimmune setting as compared with that for treating cancers.^550^ Third, most CAR-T therapeutic modalities for treating SLE are autologous CD19-targeting CAR-T cells that are limited by the cell source and thus costly.^550^ To resolve these issues, several clinical trials investigating the feasibility of allogenic CD19-targeting CAR-T cells (NCT05988216, NCT06340490, NCT05859997, NCT06429800, NCT06294236, NCT06375993)^561–566^ and allogenic CD19-targeting CAR-NK cells (NCT06421701, NCT06010472)^567,568^ in treating SLE have been registered. A pre-clinical study successfully engineered Treg cells to express CD19-specific CAR using a mice lupus model through genetic editing, which yielded promising results.^569^ Rituximab, belonging to the first generation of CD20 antibodies, has been shown with clinical and histopathological evidence in improving the symptoms of LN patients^570^ and some SLE patients refractory to conventional therapies.^495,571^ In addition, rituximab decreased the anti-dsDNA antibody levels and increased the complement levels of SLE patients according to a phase III clinical trial involving 144 SLE patients (NCT00282347) and a phase II/III study of 257 participants (NCT00137969).^572,573^ Though rituximab has not been approval for treating SLE in the clinics, it has been recommended for refractory SLE management in 2023 EULAR recommendations.^506^ To further enhance the efficacy of rituximab in depleting B cells, two phase I trials (NCT06265220, NCT06581562) have combined ribuximab with AB-101, an allogenic NK cell product capable of killing target cells via antibody-dependent cell-mediated cytotoxicity (ADCC), in 2024.^574,575^ Other CD20 antibodies with clinical evidence for SLE treatment include, e.g., ofatumumab that decreased the disease activity of 3 juvenile SLE patients in a single center study,^576^ ocrelizumab that showed efficacy in treating LN patients in a phase III study (NCT00626197),^577^ and obinutuzumab that achieved increased renal responses and decreased flares among LN patients once combined with the standard therapies in a phase II study (NCT02550652).^578,579^ CD22, though being considered as an inhibitory receptor keeping the baseline level of B cell inhibition and the humoral immunity in check, has been used as a therapeutic target for depleting dysregulated B cells due to its restrictive expression on the surface of B cells.^580^ It has been shown that epratuzumab, a humanized anti-CD22 antibody, could effectively treat moderate and severe SLE patients with acceptable safety in several phase II and phase III studies (NCT00111306, NCT00383214, NCT00383513, NCT00624351).^102,581^ But epratuzumab failed in two phase III clinical trials (NCT01262365 NCT01261793).^106^ Abatacept, a soluble fusion protein linking the extracellular domain of human cytotoxic T-lymphocyte-associated antigen 4 (CTLA-4) to the modified Fc portion of human immunoglobulin G1, can bind to CD80/CD86 on the surface of APCs including B cells for impaired adaptive immune response activation and, in particular, B cell depletion.^582^ Thus, Abatacept has been associated with reduced production of anti-dsDNA antibody and proteinuria, and increased levels of C3 and C4 levels among active class III or IV LN patients according to a phase II/III clinical trial (NCT00430677).^583–586^ However, it showed only a limited therapeutic efficacy in treating non-life-threatening SLE in a phase II study (NCT00119678) (Table 2).^585^

PCs are differentiated B lymphocyte capable of antibody secretion.^48,587^ Instead of depleting B cells, one can also remove PCs. CD38 is a pleiotropic molecule expressed on the surface of PCs.^48^ Daratumumab, being an antibody targeting CD38, has led to a dramatic desirable therapeutic response in two refractory life-threatening SLE patients, with depleted PCs among other clinical indexes being observed.^588^ Several other clinical studies investigating the efficacy and safety of daratumumab as a SLE therapeutics have been currently registered and under investigations (NCT04868838, NCT04810754). Bortezomib, being a specific, reversible inhibitor of the 20S subunit of the proteasome,^589^ significantly reduced the amount of PCs in the peripheral blood and bone marrow by approximately 50%,^590–592^ and demonstrated its treatment efficacy in dealing with refractory and severe SLE (Table 2).^589,593^

Long-lived PCs are end-staged B cells residing in the bone marrow and other tissues that can survive for decades.^594^ Long-lived PCs may drive chronic immune responses due to its sustaining production of autoantibodies,^595^ leading to persistent SLE clinical syndrome or therapeutic resistance. It is worth noting that while removing long-lived PCs without affecting their precursors may not eradicate them, depleting B cells (the precursors of PCs) may promote the development of long-lived PCs, suggesting the need of a combinatorial therapeutic design. For instance, the small-molecule proteasome inhibitor bortezomib effectively depleted PCs and reduced autoantibodies but did not affect their precursors;^596^ rituximab, the chimeric antibody against the CD20 antigen, depleted B cells but stimulated the survival of long-lived PCs and thus led to rituximab resistance.^597^ Thus, combinatorial strategies targeting both long-lived PCs and their precursors such as the ‘bortezomib+rituximab’ regimen have been considered promising in SLE treatment.^598^

Similar to the rational of depleting B cells or PCs, depleting specific subsets of autoreactive T cells associated with SLE may also contribute to SLE attenuation. For instance, immunization of 6 SLE patients with inactivated autoreactive T cells decreased the disease activities without significant side effects being observed by inducing idiotype anti-idiotypic reactions.^599^

Increasing the immune tolerance of B cells may be another attractive strategy against SLE. Abetimus sodium is an immunomodulating agent capable of inducing the immune tolerance of B cells by directly binding to and cross-linking pathogenic autoantibodies such as anti-dsDNA antibody that are pathogenetic factors of LN (a chronic kidney disease developed accompanied with SLE patients).^600^ A phase III clinical trial has indicated that administrating abetimus sodium can effectively reduce the anti-dsDNA antibody levels in LN patients (NCT00035308).^601^ Intravenous injection of immunoglobulin to neutralize the overtly produced autoantibodies has been reported as an effective therapeutics for treating LN patients including refractory LN.^602^

Though the complement system may help recognize and clear the debris to avoid autoimmune response, over-activated complement system may lead to tissue damage towards exacerbated inflammation. Thus, attenuating over-activated complement system has been proposed to treat SLE in the clinical practice. For instance, eculizumab, a C5 antibody, has been shown effective in treating complement-mediated thrombotic microangiopathy among LN and SLE patients,^603,604^ with several successful cases being reported.^605,606^ Ravulizumab, another C5 antibody, has been registered to investigate its clinical efficacy in treating LN and IgA nephropathy (NCT04564339).^607^ Avacopan, a C5a receptor inhibitor, is currently being planned to be investigated for treating SLE.^608^ Pegcetacoplan, a C3 inhibitor, has been shown effective in treating complement-mediated nephropathy including LN in a phase II study (NCT03453619)^609^ (Table 2).

### Current therapeutics restoring cytokine microenvironment homeostasis

To restore the balance of primary cytokines maintaining immune homeostasis in the microenvironment, cytokine-related therapeutics have been clinically used for SLE treatment. Specifically, administrating Th1-representative cytokines has been considered feasible for treating SLE. For instance, antibodies targeting receptors in response to Th2-generated cytokines represent an important portfolio of cytokine milieu modulating strategies for SLE treatment. For instance, IL6 promoted the maturation of B cells and differentiation of CD4^+^ T cells into Th17 but not Treg cells;^610,611^ and antibodies targeting the IL6 receptor such as tocilizumab, PF-04236921 and sirukumab showed desirable treatment efficacies in managing SLE. Specifically, the feasibility of using tocilizumab in treating SLE has been documented by a case report^612^ and a phase II study (NCT00046774);^613^ that of PF-04236921 has been reported by a post-hoc study of a phase II trial (NCT01405196);^107^ but that of sirukumab showed limited efficacies in treating CLE and SLE patients in a phase I study (NCT01702740)^614^ and among LN patients in a phase II trial (NCT01273389).^615^ It is also worth to mention that while favoring Th1 and Treg cells counterbalances the skewed immune microenviroment of SLE patients, it may revert the cytokine profile to the other end, rendering dual targeting of opposite cytokine axes towards microenvironment homeostasis an emerging trend for SLE therapeutics. For example, ustekinumab,^614,615^ a monoclonal antibody neutralizing the activities of IL12 (facilitating Th1 development) and IL23 (secreted by Th17 cells), has been show effective and safe in treating SLE from a two-year phase II study (NCT02349061).^616,617^ As another example, inhibitors of phosphodiesterase 4 (PDE4) such as apremilast have been shown effective in treating autoimmune diseases including refractory skin lesions of lupus in a trial involving 5 participants^618^ and in a phase II study involving 8 CLE patients (NCT00708916)^619^ by suppressing Th1 and Th17-mediated immune responses^620^ (Table 2).

Restoring Th17/Treg homeostasis via directly injecting cell materials has been proposed as a promising strategy for treating SLE. For instance, Tregs injected into lupus mice has attenuated the inflammatory response and alleviated the pathological syndromes of SLE.^621^ Stem cell therapies have shown a long-term efficacy and safety for SLE management^622–624^ by increasing the proportion of Treg cells among SLE patients.^625^ Low-dose IL2 has been associated with a rapid remission of SLE or CLE with good tolerance by rewiring the skewed Th1/Th2 cytokine microenvironment from several clinical trials of phase II or unavailable (NCT02955615, NCT02465580, NCT02932137, NCT02084238).^626–631^ Supply of IL10 antibody (that can be naturally produced by Treg cells) has been documented to be feasible in treating refractory SLE patients according to a clinical study involving 6 participants.^632^ From another point of view, secukinumab, an IL17A antibody functioning via blocking the interactions between IL17A and its receptor, has been successfully used to treat multiple autoimmune disorders such as ankylosing spondylitis, psoriasis, leprosy and,^633,634^ importantly, refractory LN patients.^635–637^ Pre-clinical studies have also shown that exosomes from umbilical cord blood MSCs can restore the Th17/Treg balance by lowering the percentage of Th17 subsets (Table 2).^638^

Some hormonal therapies have been shown effective in treating SLE via modulating the immuno-regulatory milieu. Glucocorticoids such as betamethasone,^639^ dexamethasone,^640^ hydrocortisone,^641^ prednisone,^642^ prednisolone,^643^ triamcinolone,^644,645^ methylprednisolone,^393,646,647^ which have long been applied topically or systematically for SLE treatment, are prone to inhibit the differentiation of Th17 cells and enhance that of Treg cells. In particular, glucocorticoids can decrease the number of Th17 cells and lower the level of IL17A for attenuated SLE symptom,^648^ and requires the aid of functioned Treg cells to exert anti-inflammation roles^649^ as evidenced by the upregulated Treg cells on glucocorticoid intake.^650^ Interestingly, increased levels of systemic glucocorticoid may protect the organism from running into Th1 over-dominance by modulating the Th1/Th2 balance, where the expression of Th1­promoting cytokines such as IL12 and CSF were down-regulated and the secretion of Th2­type cytokines such as IL4, IL10 and IL13 were up-regulated.^651,652^ Also, methylprednisolone pulse therapy has demonstrated its feasibility in treating SLE via promoting Treg cell differentiation (Table 2).^647^

Metabolic abnormalities remain as the underlying mechanism of Th17/Treg imbalance in SLE, as Th17 and Treg cells bear different metabolic patterns. In particular, while glycolysis, pentose phosphate pathway, fatty acid synthesis, and glutaminolysis are predominant in Th17 cells, fatty acid oxidation and oxidative phosphorylation are active in Treg cells. Thus, reprograming T cell metabolic patterns has emerged as an effective strategy for managing SLE patients through reversing the Treg/Th17 imbalance and restoring the immune homeostasis.^653^ Following this rational, rapamycin, a mTOR inhibitor, has been proposed for SLE treatment that functions by reprogramming T cell metabolic patterns towards prevented over-activation of Th17 cells and improved Treg proliferation, and been proven to alleviate disease severity among SLE patients in a phase I/II trial (NCT00779194).^654,655^ Statins, a class of drugs that inhibit cholesterol biosynthesis, have been reported capable of targeting Th17/Treg imbalance and alleviating Th17-mediated inflammatory response.^656^ For example, atorvastatin reduced atherosclerosis progression in lupus patients according to a phase III study (NCT00065806),^657^ and improved arterial stiffness and decreased SLE disease activity in an 8-week clinical study involving 37 SLE females;^658^ pravastatin decreased the total cholesterol and low density lipoprotein (LDL) levels in SLE patients;^659^ rosuvastatin reduced the lipid levels of SLE patients;^660^ and simvastatin decreased the levels of antiphospholipd antibodies among SLE patients.^661^ Besides, replacing glucose with galactose and supplementing 2-Deoxy-D-glucose (2-DG, an inhibitor of hexokinase that functions as the first rate-limiting enzyme of glycolysis) all diminished Th17 development and enhanced Treg differentiation^78–80^ (Table 2).

### Current therapeutics rescuing impaired debris clearance machinery

SLE patients are characterized by high autoantibody loads that form ICs, inefficient clearance of which accelerate SLE syndroms. Most autoantibodies found in SLE carriers are IgG that can be recognized by FcγRIIIB, a type of Fcγ receptors capable of recognizing the Fc portion of IgG and expressed on neutrophils and a subset of basophils. FcγRIIIB participates in IC clearance by promoting phagocytosis, adhesion, and the respiratory burst in neutrophils.^662^ FcγRIIB is another member of the FcγR family that are primarily expressed on B cells, macrophages, and DCs.^663^ As dysfunctional FcγRIIB and FcγRIIIB have both been implicated in priming SLE,^662^ therapeutics mimicking the effect of these receptors have been established for SLE management. For instance, **v**alziflocept, a recombinant soluble human FcγRIIb that binds to ICs as a decoy, has been shown promising in a double-blind placebo-controlled multicenter study for managing SLE symptoms.^663,664^

Purified or recombinant complement proteins may not be recommended for treating SLE in the clinics due to the possible damaging effects of overtly stimulated complement system. Specifically, hyperactivated complement system via the alternative pathway may damage cells such as podocytes and tissues such as the blood vessel endothelium that deteriorate the SLE symptoms. Thus, the fresh frozen plasma containing multiple complement proteins has been launched in the clinics, with therapeutic successes being documented in treating *C1q*-deficient SLE patients^665^ and those harboring *C2* deficiency.^666^ Plasma exchange may represent another strategy to fuel sufficient complements in the blood to remove pathological antibodies and their complexes with the immune components in time for alleviated SLE symptoms (Table 2).^667–669^

## Conclusions

We reviewed the history of the SLE research field and the epidemiology of this disease, grouped the mechanisms-of-action driving SLE pathogenesis into three categories, i.e., activating the immune response, skewing the cytokine microenvironment, and impairing the debris clearance machinery; summarized current knowledge on SLE diagnosis by the disease onset, activity and comorbidities; identified risk factors predisposing SLE at the genetic, epigenetic, hormonal, extrinsic levels; and, importantly, classified current SLE preventive and treatment strategies following the logic of the identified mechanisms. It is worth noting that these groups used for classifying current SLE preventive and therapeutic strategies are not mutually exclusive, as most SLE management approaches take action through multiple mechanisms. For instance, glucocorticoid, primarily functions via modulating the cytokine microenvironment,^648–652^ also attenuates the immune response by inhibiting the maturation and activity of DCs, interfering with TCR signaling, and inducing B cell apoptosis as well as affecting the downstream pathways of B cell receptor signaling such as NF-κB.^349^ As another example, the methylprednisolone pulse therapy functions not only by promoting Treg cell differentiation, but also via inducing the apoptosis of CD4^+^ T cells.^647^ Similarly, hydroxychloroquine, known capable of alleviating SLE and largely by blocking TLR signaling,^455^ also modulates the cytokine distribution and homeostasis among Th1, Th2, Th17 and Treg cells.^670,671^

Given our incremental understanding on the priming role of intrinsic features in predisposing SLE and the increasing need for precision medicine, focusing on personalized SLE treatment taking advantages of genetic or immunological markers may represent a promising therapeutic perspective. Thus, identifying representative markers associated with each of the three identified pathogenesis stages and establishing rapid yet cost-effective screening techniques may aid in the therapeutic design and lead one future direction.
